# Design and fabrication of graphene oxide-based nanocomposites with tuneable morphological properties for advanced supercapacitor applications

**DOI:** 10.1039/d6ra06017j

**Published:** 2026-10-16

**Authors:** Mohammad Mizanur Rahman Khan, Md Mahamudul Hasan Rumon, Jae-Ho Jeong

**Affiliations:** a Department of Chemistry, Shahjalal University of Science and Technology Sylhet-3114 Bangladesh mmrkhan-che@sust.edu; b Department of Chemistry, Bangladesh University of Engineering and Technology Dhaka 1000 Bangladesh; c School of Mechanical Engineering, College of Engineering, Chung-Ang University 84 Heukseok-Ro, Dongjak-Gu Seoul 06974 Republic of Korea jaehojeong@cau.ac.kr

## Abstract

The quicker progress of renewable energy conversion and storage technologies has resulted in devices that are more efficient and operate steadily, thus helping in alleviation of global energy crisis. Although much research is still underway on GO-based electrodes for supercapacitors, their performance is not yet suitable for commercial implementation. There is still a knowledge gap regarding the morphological characteristics, synthesis, and design of appropriate materials for supercapacitor applications. The current review particularly highlights the synthesis approaches and morphological features of GO-based composites and their use as electrodes for high-performance supercapacitors (HPSs). We discuss in detail the synthesis methods and their associated mechanisms, as well as the electrochemical methods used to fabricate supercapacitors (SCs). Most importantly, the electrochemical performance and potential applications of these GO-based nanocomposites in SCs are explored. This review also highlights the recent advances in GO-based nanocomposites incorporated with metal-based materials and polymers, along with their corresponding electrochemical performance in SCs. Large-scale applications require HPSs, and this review provides a comprehensive overview that offers valuable insights into the applicability of GO-based nanocomposites in HPSs.

## Abstract 2

Synthesis strategies for GO-based nanocomposites for supercapacitor applications, highlighting key performance metrics through morphology control, scalable synthesis, and optimized material design.

## Graphical abstract

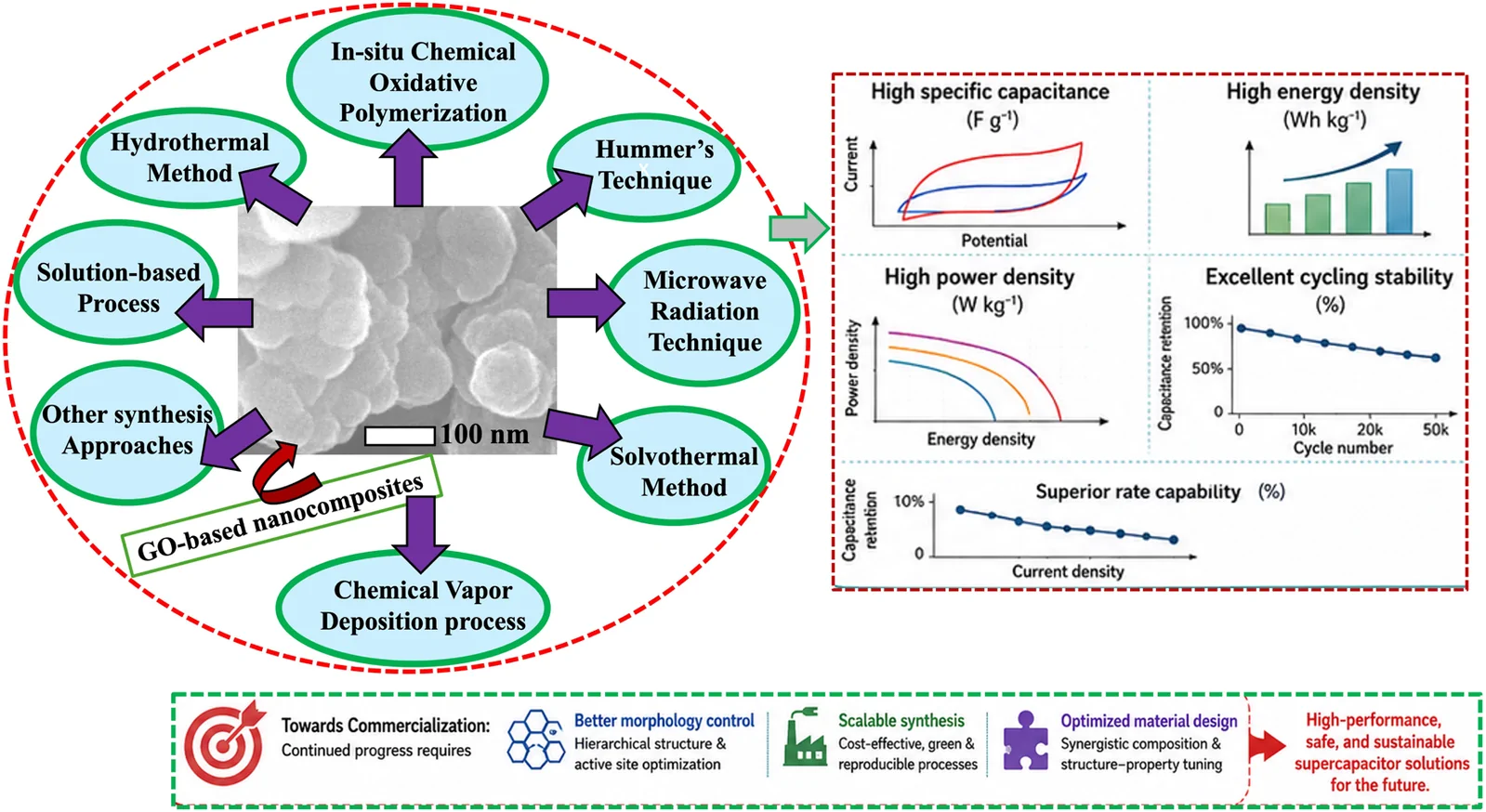

## 1 Introduction

The increasing global demand for energy raises concerns about social well-being, climate change, and sustainable human development.^1,2^ To meet the rising energy demand, energy storage technologies can provide an effective solution by regulating energy discharge based on demand and generation requirements. In this context, capacitors can store energy by accumulating charges and creating a potential difference across plates. Nevertheless, low dielectric constants limit their energy density. This limitation has driven researchers to explore supercapacitors (SCs), which offer a special combination of exceptional power density, extended cycle life, and rapid charge/discharge rates.^3–5^ Moreover, SCs are being increasingly used as green energy storage devices owing to their environmental friendliness, memory backup capabilities, and low maintenance needs. These characteristics of supercapacitors have gained a lot of interest in recent years.^6,7^ Further, these significant features make supercapacitors (SCs) ideal for a range of uses, such as power electronics and power amplifiers, where rapid energy discharges and frequent cycling are required. However, SCs have drawbacks like limited energy density, as reported by researchers.^8,9^

To address the shortcomings of typical SCs, researchers have proposed a variety of alternatives based on material synthesis, morphology and configuration. Reported works in this field include investigations aimed at enhancing the electrochemical performance of the electrode for the creation of smart SCs in energy storage applications.^10–12^ Another drawback is the poor specific energy of SCs, which limits their commercialization.^13,14^ Therefore, it is necessary to design and fabricate prospective electrode materials to enhance the specific energy of SCs. Many types of materials, including CPs^15,16^ and carbon materials,^17^ have been investigated for SC electrodes in recent years. Thus, significant attempts are required to develop suitable materials for advancements in the formation of high-performance SCs. In this regard, GO-based composites are gaining significant attention among researchers owing to their distinct blend of properties. This synergistic arrangement allows definite control over ion transport kinetics and improves the electrochemical properties. Because graphene and GO only store energy by electrostatic adsorption and desorption, they have certain drawbacks, including low capacitance and lower energy density. In order to get exceptional results for supercapacitor applications, researchers were motivated to develop composites using graphene and GO with other potential materials, which resulted in several hybrid composites.^18,19^ In this context, a wide range of metallic and nonmetallic materials with moderate electrical conductivity, such as conducting polymers and metal oxides, can be used with GO to create effective electrodes for SCs.^20^ Furthermore, the morphology of GO-based composites can be tailored to improve supercapacitor performance.^21,22^ There are numerous advanced elements, including metal oxides, CPs, and so on, which have amazing features as electrodes for supercapacitor application.^22–24^

To date, numerous studies have reported the design and fabrication of graphene- and graphene oxide (GO)-based composites as electrode materials for next-generation supercapacitors.^25,26^ These studies have explored strategies to tailor the composition and morphology of graphene-based composites^24^ and demonstrated their potential for energy-storage applications.^27^ In addition, graphene-based composites have shown considerable promise in a wide range of applications, including in Li-ion batteries, supercapacitors, fuel cells, photovoltaic devices, and photocatalysis.^28^ However, despite these advances, systematic studies correlating the morphological features of GO-based nanocomposites with their electrochemical performance in supercapacitors remain limited.

Further, the performance of a supercapacitor can be impacted by the fabrication method of the electrode materials. Hence, to meet the current and upcoming demands, energy storage systems must be more effectively developed, manufactured, and evaluated. To improve performance, the morphology of the electrode materials used in SCs can also be altered.^29,30^ The total performance of SCs can be enhanced by combining materials into a single electrode. Among the cutting-edge materials that demonstrate exceptional properties as electrodes for supercapacitor applications are metal-based materials and conducting polymers (CPs).^31,32^ To overcome the current shortcomings of SCs, it is necessary to investigate the distinct gaps in the synthesis process, the morphology of the prepared GO-based composites, and their successful applications as perfect electrode materials for high-performance SCs.

Accordingly, the goal of the current review is to outline the recent advancements in the manufacturing of GO-based composites for use in supercapacitors. It explores the developments in the design and synthesis of GO-based electrodes for HPSs through an in-depth discussion. The methods used to evaluate the electrochemical performance are also discussed. Here, we also provide details of the latest study on various synthesis methods, their importance, and the morphology of these GO-based composites as electrode materials. These paths have the potential to not only solve the present issues but also open the possibility for the further development of supercapacitor technologies, guaranteeing their applicability and effectiveness in changing energy storage environments.

## 2 Synthesis of GO-based nanocomposites

GO is combined with other materials to form GO-based nanocomposites with improved properties. Graphite is first chemically oxidized *in situ* to give GO using the Hummers' method. It is then combined with other materials using techniques including mixing, intercalation, and *in situ* polymerization. GO is a flexible component used for synthesizing nanocomposites with enhanced performance that can be used in fields like energy storage due to its significant surface area and oxygenated functional groups. Numerous synthesis strategies for fabricating GO-based nanocomposites have been reported in the literature, including hydrothermal and solvothermal methods, solution-based processes, and microwave radiation techniques(Fig. 1). These synthesis approaches and their details, advantages and disadvantages are listed in Table 1. The promising synthesis approaches for the GO-based nanocomposites are discussed in this section.

**Fig. 1:**
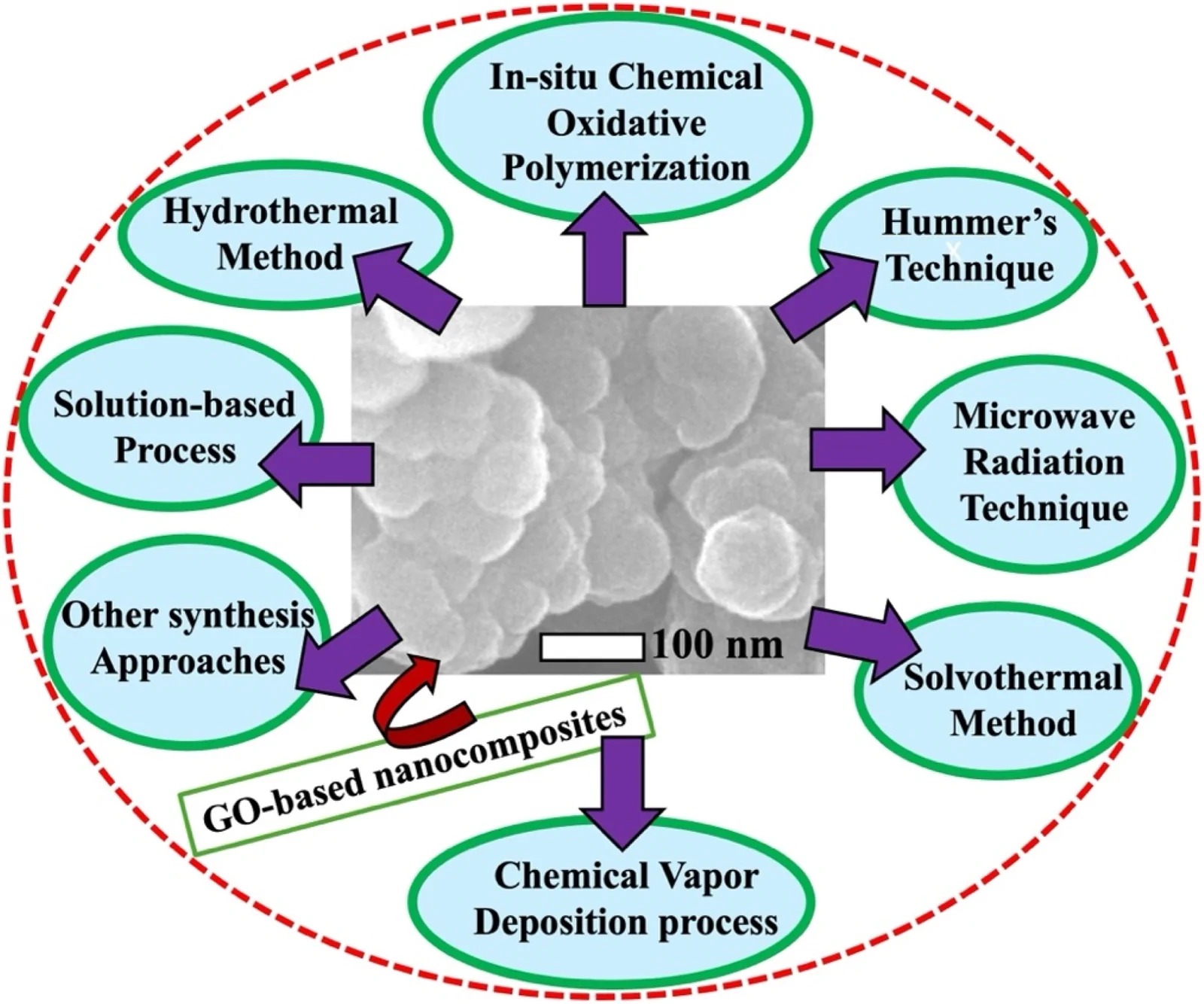
Numerous synthesis strategies of the GO-based nanocomposites.

**Table 1:** Details of numerous synthesis approaches for GO-based composites along with the advantages and disadvantages of each synthesis process

Synthesis approaches	Details	Advantages	Disadvantages	Ref.
Hummers' technique followed by plasma treatment	Stirring, functionalized with dielectric barrier discharge plasma reactor at ambient pressure	Easy strategy to fabricate functionalized materials; large-scale synthesis	Complexity of plasma processes	33 and 34
Modified Hummers' process	Maintained low temperature in the synthesis	Possible to synthesize carbon-based materials, time-saving approach	Low purity, costly process	35 and 36
*In situ* chemical oxidative approach	Direct approach, need oxidizing agents, ultrasonication, autoclave reactor	Cost-effective, and green approach	Complex treatment	37–39
Hydrothermal technique	Ultrasonication for dispersion of GO	Possible to attain good structural stability and electrochemical behavior	Complicated experimental setups, challenging to control the growth process	40–43
Microwave radiation-assisted technique	Stirring, ultrasonication, domestic microwave	Simple, fast and easy process, controllable synthesis, and cost-effective	Required certain and expensive equipment, complications in scaling up the process, defects in material structure, unexpected side reactions	44–46
Solvothermal process	Non-aqueous solvent, high temperature and pressure	Controllable synthesis	Lengthy reaction times, probabilities of GO aggregation	47–49
Solution-based process	Mixing GO with another material	Able to attain uniform dispersion, processing requires low temperature	Aggregation of GO, and complications in removing impurities	50 and 51
Chemical vapor deposition process	Ultrasonically washed with solvent, dried using vacuum oven	Superior materials, scalability, possibility of green synthesis, and versatile material design	Low yield, time-consuming technique, and expensive process	52 and 53

### 2.1. Hummers' technique

A popular chemical oxidation technique used for synthesizing GO from graphite is the Hummers' method. In this process, KMnO_4_ and concentrated H_2_SO_4_ are utilized to oxidize graphite flakes to produce GO. Generally, after synthesizing GO by the Hummers' technique,^54^ researchers utilized other fabrication methods for the formation of numerous GO-based nanocomposites.^55^ For example, Sim *et al.* fabricated fluorine-functionalized GO composites through a plasma treatment process.^33^ The synthesized GO-based composites were applied for supercapacitors. In another study, GO was formed through the modified Hummers' method, where graphite was oxidized utilizing KMnO_4_, NaNO_3_, and H_2_SO_4_.^56^ Here, graphite powder enlarges the interlayer arrangement of carbon sources. Afterwards, GO-Ag nanocomposites were fabricated by mixing GO and Ag nanoparticles in a 2 : 1 ratio using methanol as a solvent, followed by sonication for 2 days.^56^ MnFe_2_O_4_/GO nanocomposites were prepared using the modified Hummers' method at low temperatures.^35^ Further, utilizing this process, it is easy to synthesize carbon-based materials, thereby saving time.

### 2.2. *In situ* chemical oxidation approach

*In situ* chemical oxidation approach is an important technique to obtain GO-based nanocomposites. In this process, an oxidant (for example, ammonium persulfate) is often used in the presence of GO to form a GO-polymer nanocomposite, as presented in Fig. 2. Yildiz *et al.* recently synthesized a type of rGO-Au@PANI nanocomposite following a simple two-step approach.^37^ This method is also a cost-effective and green approach. Thin film-like electrode materials are formed on nanosized rGO sheets, which were coated with very fine polymeric layers. The Au nanoparticles were prepared within the nanocomposite films with diameters ranging from 5 to 20 nm.^37^ Basu *et al.* prepared PANI/GO/MoS_2_ nanocomposites through an oxidative polymerization technique and studied their morphology, such as surface area and porosity, in detail.^57^ At first, GO/MoS_2_ was prepared utilizing MoS_2_ as a precursor *via* an *in situ* approach. Then, PANI/GO/MoS_2_ ternary nanocomposites were synthesized using 1 : 1 GO/MoS_2_ through the oxidative polymerization technique.^57^ The prepared nanocomposites exhibited a higher surface area than that of PANI, GO, and MoS_2_, indicating their enlarged porosity.

**Fig. 2:**
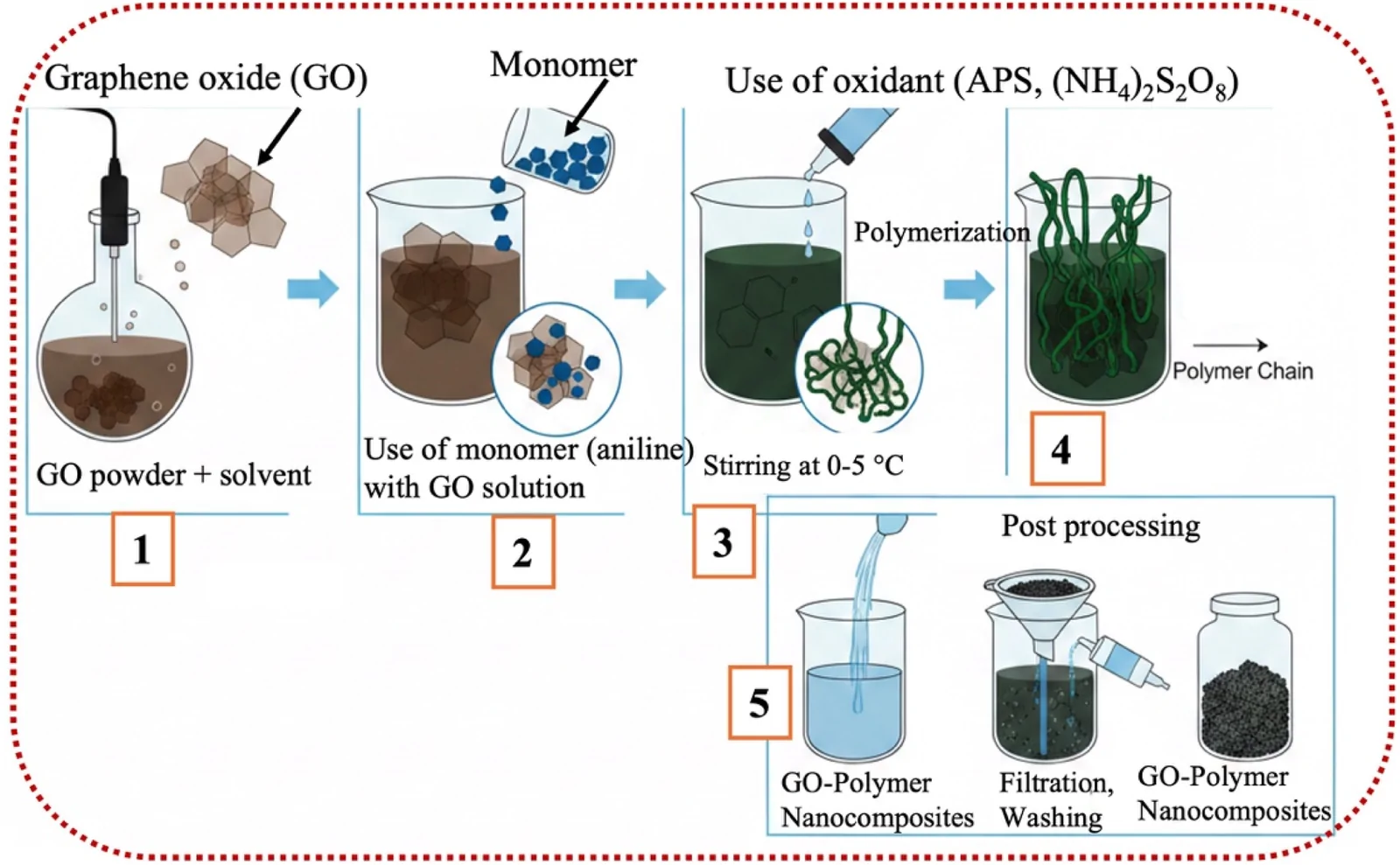
Schematic of GO-based polymer nanocomposite formation *via* an *in situ* chemical oxidative polymerization process.

Polypropylene (PPy)/GO nanocomposites were fabricated by *in situ* Ziegler–Natta polymerization.^38^ In this case, the catalysts were supported by GO. First, the Grignard reagent, C_4_H_9_MgCl (BuMgCl), was synthesized from chlorobutane and magnesium powder under an N_2_ atmosphere at 80 °C for 20 h under reflux.^38^ After mechanical stirring, the polymerization reaction was performed in an autoclave reactor, which was initiated by adding triethyl aluminium and dimethoxydiphenylsilane to the reactor.^38^ This reaction is the first example of the synthesis of PPy/GO nanocomposites through *in situ* polymerization utilizing the Ziegler–Natta catalyst.

GO/PANI/PVA and rGO/PANI/PVA were fabricated *via* an *in situ* oxidative polymerization approach.^39^ The prepared nanocomposites are additive-free and can act as bifunctional electrodes for supercapacitor applications. The reported method is an innovative and easy polymerization strategy to prepare GO-based nanocomposites under mild conditions. This approach utilized 100% active material for the fabrication of electrodes, overcoming the limitations generally faced in energy storage and conversion applications.^39^

### 2.3. Hydrothermal method

A typical technique for the formation of GO nanocomposites is hydrothermal synthesis. In this approach, GO is reacted with other materials at high temperatures (usually 120–220 °C) in a closed, pressurized system to promote the chemical reactions that result in the formation of nanocomposites. The capacity to produce crystalline phases that are unstable at their melting temperatures is a significant merit of hydrothermal synthesis. The convenient fabrication of nickel ferrite/graphene nanocomposites utilizing the hydrothermal process was reported in a previous study.^40^ Balu *et al.*^58^ fabricated SnS/graphene nanocomposites as electroactive materials for HPSs through a hydrothermal approach. In contrast, the coating of GO flakes may have reduced the carboxyl functional group peaks, which was also confirmed by XRD data. In the synthesis process, GO was ultrasonically dispersed, which may facilitate the formation of SnS-decorated SnS/graphene nanocomposites.^58^ A hydrothermal approach was used for the synthesis of MnCoS/rGO composites with superior supercapacitor performances by E. Manikandan *et al.*.^59^Fig. 3 presents the schematic of the hydrothermal synthesis approach for the formation of MnCoS and MnCoS/rGO composites. Generally, the synthesis of sulfide-based materials is a complicated and expensive approach that needs extreme temperatures and an inert atmosphere. In the work, the authors successfully prepared sulfide-based nanocomposites with rGO that demonstrated ideal electrochemical properties for supercapacitors.

**Fig. 3:**
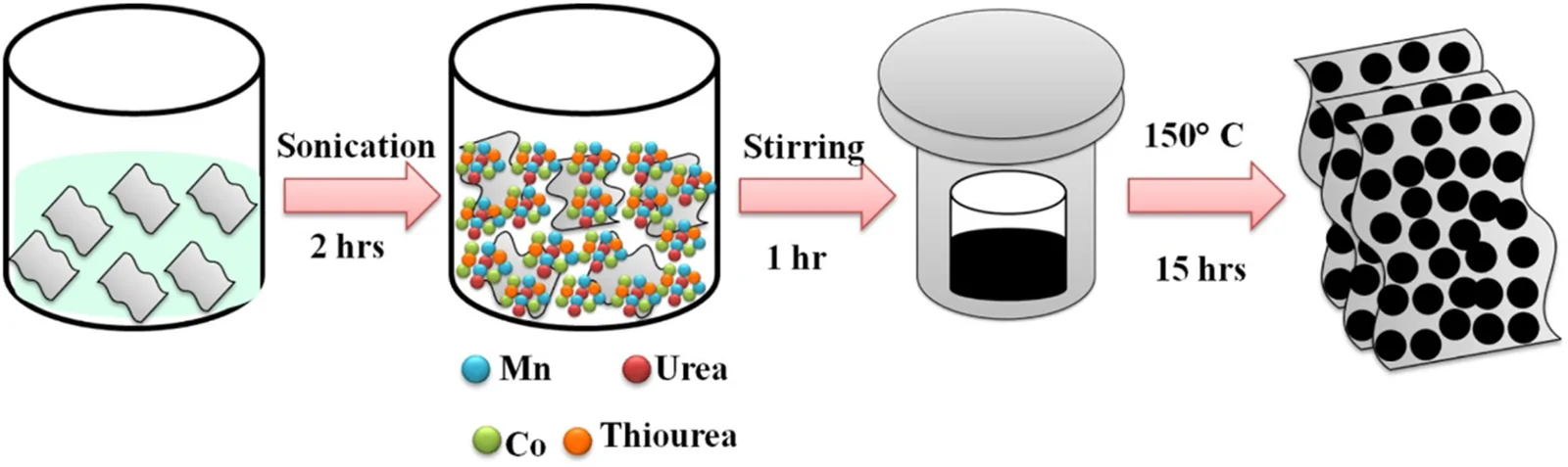
Schematic of the synthesis of the MnCoS and MnCoS/rGO composites using a hydrothermal process. Reproduced with permission from ref. 59 Copyright 2024, the author(s).

Min Fu *et al.* synthesized 1D nickel ferrite/graphene nanocomposites for supercapacitor electrodes utilizing a one-step hydrothermal process.^40^ The authors demonstrated that the growth of nickel ferrites on graphene improves its structural stability and provides good electrochemical features. The hydrothermal route was also utilized for the fabrication of δ-MnO_2_/N-doped rGO composites for supercapacitor performance.^60^ In the synthesis process, *p*-phenylenediamine acts as the “anchoring molecule” and nitrogen source for the N-doped composites. The uniform nanostructures of MnO_2_ were also found to grow using this synthesis technique.^60^ In another study, hollow-structured NiCo_2_O_4_ nanorod composites with N-doped graphene were fabricated for HPSs.^61^ Using this process, the modified 3D-structured graphene with a high nitrogen content can increase the active sites as well as the surface area of the prepared nanorod composites.^61^ The rGO-based MnO_2_ nanocomposites synthesized by the same procedure exhibited superior electrochemical properties for supercapacitor applications, as reported by Gosavi *et al.*.^62^ Here, MnO_2_ plays a vital role as an active component for the electrode materials utilized in supercapacitors. The sonication process was used to generate the homogeneous mixture needed for composite preparation.^62^ Another potential ternary nanocomposite of polypyrrole/rGO/ferrites with good electrochemical properties was prepared by a hydrothermal approach.^63^ The synthesis process was conducted using a Teflon-lined autoclave, maintaining a temperature of 180 °C for 12 h.^63^

### 2.4. Microwave radiation-assisted technique

Microwave-assisted synthesis is a technique utilized for the formation of GO-based nanocomposites. This method has several advantages, including rapid and efficient heating, and cost-effectiveness. It enables chemical processes *via* microwave radiation, aligning dipoles in the material and speeding up the synthesis process. However, this approach has numerous drawbacks, including scale-up complications, non-uniform heating, the formation of structural defects, and unexpected side reactions during synthesis. Utilizing the microwave irradiation-assisted process, Matsuda *et al.* synthesized homogeneous NiO-MnO_2_@rGO composites with excellent electrochemical properties for high-performance supercapacitors.^44^ It was a fast and simple approach to obtain ternary homogeneous composites like NiO-MnO_2_@rGO. A GO-based magnetic nanocomposite (M/GO) was also prepared using microwave irradiation with a good surface area (126 m^2^ g^−1^).^64^ The achieved surface area provides an abundant number of active sites within composite matrix, which may be useful for their successful applications. In another study, SnO_2_:rGO nanocomposites were synthesized utilizing a microwave-assisted hydrothermal technique with the modification of material properties such as optical, electrical, and morphological features.^65^ During the synthesis process, a homogeneous solution was obtained by ultrasonic stirring, and the gel-form mixture was heated at 700 W with 2200 MHz.^65^ Tsai *et al.* fabricated PtNiCo/rGO composites *via* a one-step microwave-assisted approach, demonstrating excellent electrochemical performance that renders them suitable as energy storage materials.^66^ For their synthesis, a homogeneous dark brown solution was adjusted to alkaline pH (pH 10), and the resulting solution was heated at 200 °C for 20 min. A narrow particle size distribution was identified for Pt-Ni-Co nanoparticles homogeneously dispersed in rGO without agglomeration. The authors also claimed that the prepared PtNiCo/rGO composites have superior electrocatalytic activities, including a wide electrochemically active surface area and high electrochemical stability.^66^

Very recently, K.-Y Shin *et al.* have also synthesised MnFe_2_O_4_/rGO nanocomposites using a one-step microwave-assisted technique suitable for supercapacitor applications.^67^ This approach was rapid compared to the other conventional hydrothermal approaches. In this process, the prepared suspension was transferred into a Teflon-lined reactor, followed by microwave heating with a power setting of 800 W, essential for the synthesis of MnFe_2_O_4_/rGO nanocomposites (Fig. 4a). The formation method of the composites relies on the electrostatic attraction between the positively charged Mn^2+^ and Fe^2+^ ions and the negatively charged GO surface. Under high-temperature conditions, Fe^2+^ is oxidized to Fe^3+^ on the GO surface, concurrently inhibiting the oxidation of Mn^2+^. In this reaction, NaOH promotes the deposition of metal particles onto the rGO surface. Moreover, rGO establishes a resilient supporting matrix that facilitates the adherence of MnFe_2_O_4_ between the rGO layers. The overall experimental arrangement of this approach is presented in Fig. 4b.

**Fig. 4:**
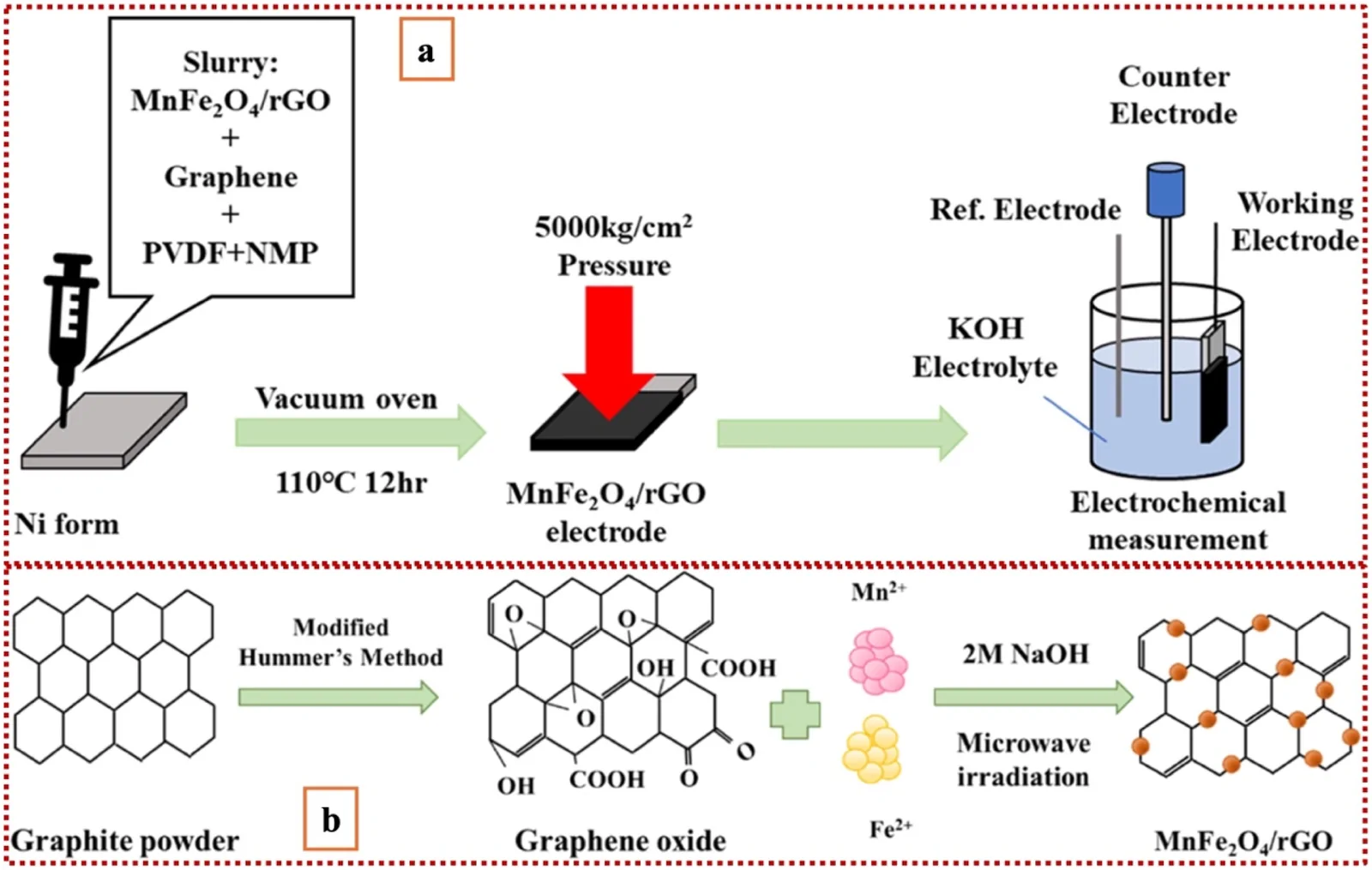
(a) Schematic of the formation of MnFe_2_O_4_/rGO nanocomposites. (b) Experimental arrangement for the nanocomposite preparation. Reproduced with permission from ref. 67. Copyright 2025, the American Chemical Society.

### 2.5. Solvothermal method

GO-based nanocomposites can be synthesized using a solvothermal approach, where the process involves using a non-aqueous solvent to dissolve the reactant. The process also involves high-temperature and high-pressure conditions to enable the reaction and the formation of expected nanocomposites. The MnFe_2_O_4_ quantum dots/nitrogen-doped graphene were fabricated through a solvothermal process with excellent electrochemical properties that are very useful as an electrode for HPSs.^47^ The fabricated materials are homogeneously dispersed on nitrogen-doped graphene surfaces, where GO plays a vital role in the general structure.^47^ Lanthanide (Ln)-functionalized GO nanocomposites were fabricated utilizing a solvothermal technique, as reported by Basiuk *et al.*.^48^ The formation mechanism of the composites is assumed to be the coordination of Ln^3+^ ions to the oxygen-containing functional groups of GO, which serve as the nucleation sites, and the nanocomposites maintain the planar honeycomb-like structures similar to graphene.^48^ Ojha *et al.* prepared 2H-WSe_2_@rGO nanocomposites following the controlled synthesis process, such as the solvothermal technique.^68^ The synthesis process involves the use of reducing agents like NaBH_4_ as well as specialized equipment such as Teflon-lined stainless steel autoclaves and muffle furnace operating at 200 °C for 46 h.^68^ There is another report on the efficient synthesis of ZrO_2_/rGO nanocomposites (NCs) through the solvothermal technique.^69^ This is a one-pot green synthesis process, where ZrO_2_ was homogeneously dispersed on the rough surface of rGO. The schematic of the synthesis route is displayed in Fig. 5.

**Fig. 5:**
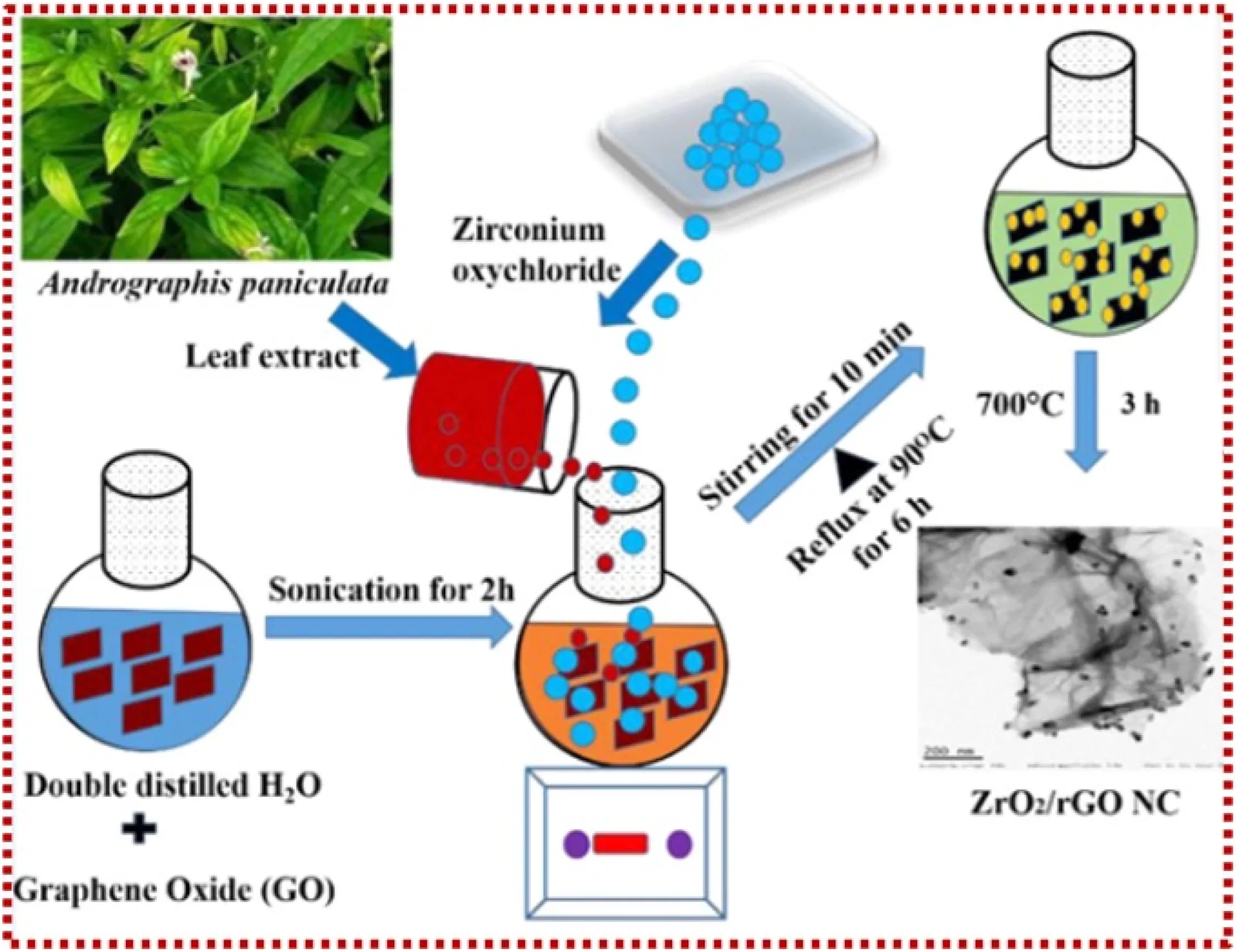
Schematic of the synthesis route of the ZrO_2_/rGO nanocomposites (NCs). Reproduced with permission from ref. 69. Copyright 2023, the American Chemical Society.

### 2.6. Solution-based process

In the solution-based process, nanocomposites are formed by mixing GO with another material in a solution, following other processing steps. In this process, uniform dispersion of the materials is possible, and it is a low-temperature process to obtain composites. For example, Soam *et al.* synthesized nickel ferrite (NiFe_2_O_4_)/graphene nanocomposites through the solution-based process, exhibiting high electrochemical performance.^50^ This synthesis process is very useful to enhance the supercapacitor performance of the composite materials. The interlinked pores in the electrode and favorable electrical contact of NiFe_2_O_4_ enable the migration of more ions from the electrolyte and ultimately enhance the supercapacitor performance.^50^ Another work is reported in the literature on the preparation of RGO-poly(methyl methacrylate)(PMMA) nanocomposites by a solution intercalation approach for supercapacitors.^70^ A schematic of this approach is displayed in Fig. 6. The authors claimed that the obtained outcomes are promising for utilizing the prepared nanocomposites for the fabrication of supercapacitors.^70^ Although the solution-based process has potential, there are limited works reported in the literature. Therefore, there are opportunities to explore this technique to find suitable GO-based nanocomposites for HPSs.

**Fig. 6:**
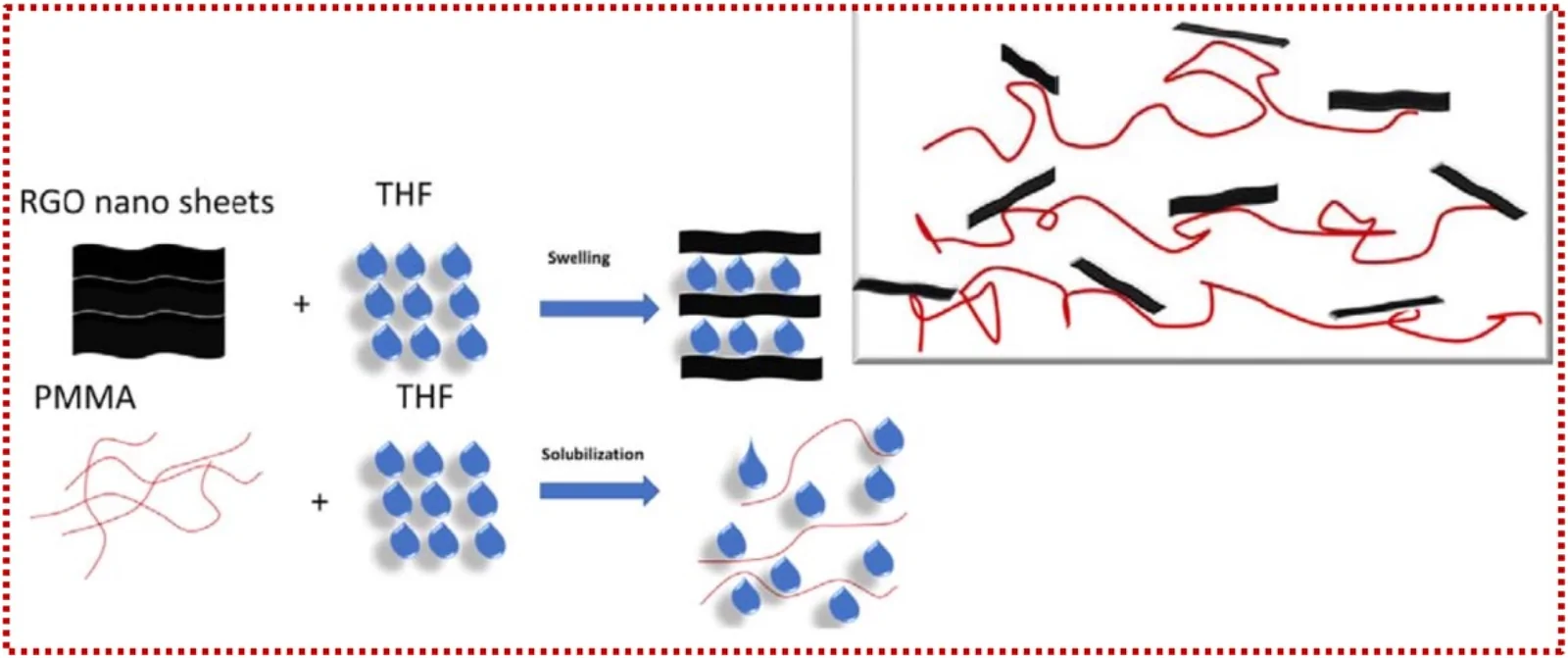
Design of the synthesis route of the RGO/PMMA nanocomposites. Reproduced with permission from ref. 70. Copyright 2018, Wiley Periodicals, Inc.

### 2.7. Chemical vapor deposition process

Chemical vapor deposition (CVD) has several advantages, such as improved electrochemical performance, enhanced electron transport, and inexpensive manufacturing; thus, it is beneficial for the synthesis of GO-based nanocomposites for supercapacitors. To obtain high-quality, low-defect graphene, the CVD technique is usually applied, which aids in optimizing the capacitance. Additionally, by integrating GO with other materials and facilitating the production of three-dimensional structures, the technique improves the stability and performance of the final nanocomposites. To obtain a stable electrode with a favorable chemical stoichiometry, chemical vapor deposition may serve as an excellent method for the synthesis of network-like composites with supercapacitor performance. For example, Ni_3_S_2_/3D graphene/nickel foam-type composites were synthesized utilizing a chemical vapor deposition approach for electrochemical applications.^52^ The characteristics of the flawless heazlewoodite stage of Ni_3_S_2_, the larger specific area of the 3D graphene, and the foam substrate of Ni facilitate the supercapacitor performance of the prepared composites.^52^ Cobalt core/graphite shell nanostructures were synthesized utilizing a chemical vapor deposition approach with ferromagnetic properties.^71^ This is a one-step synthesis process, where the prepared cobalt core/graphite nanostructures were anchored onto the graphene sheets.^71^

### 2.8. Other synthesis approaches

Besides the above-mentioned synthesis methods, there are numerous other synthesis techniques to synthesize GO-based nanocomposites for supercapacitor applications. These include multi-step thermal processes,^72^ annealing-assisted sol–gel techniques,^73^ co-precipitation methods,^74^ and electrodeposition processes.^75^ Using the multi-step thermal process, Lv *et al.* synthesized a sandwich-type structure of graphene and NiO from the precursors of GO-Ni(NO_3_)_2_ for high-performance supercapacitors.^72^ This structure avoids aggregation during electrochemical charge–discharge cycling, maintains the active surface area, facilitates open channels for ion transport.^72^ Hydrous RuO_2_/graphene composites were fabricated utilizing both sol–gel and low-temperature annealing processes.^73^ In this process, chemically exfoliated graphene sheets serve as an anchor for the RuO_2_ particles on the surface. The formation of RuO_2_/graphene sheet composite structures provides excellent electrochemical properties for supercapacitor applications.^73^

Using an electrostatic co-precipitation technique, MnO_2_ nanosheets were applied to the functionalized rGO to obtain their composite material.^74^ The fabricated composites demonstrated improved capacitive performance with excellent cycling stability desirable for supercapacitor applications. The layered structure of the prepared composites endows them with good capacitive performance. The electrodeposition process is another reported approach to obtain HPSs using graphene hydrogel and MnO_2_ in the nanostructure form.^75^ In this work, first, GO was prepared from graphite powder, and a graphene hydrogel was prepared utilizing l-glutathione, which acts as a reducing agent in the reaction. In this reaction, the excess amount of l-glutathione was removed following the dialysis process.^75^ El-Shafai *et al.* prepared GO@TiO_2_ nanocomposites using the sol–gel process.^75^ Other metal oxides (MO) like MgO, SiO_2_, Co_3_O_4_, and V_4_O_7_ were doped with the composites for supercapacitor applications. In the fabrication process, grinding and ultrasonication were performed to obtain the desired GO@TiO_2_-MO composites.

For use in supercapacitors, a NiO/MnO_2_/GO composite was fabricated utilizing NiO, MnO_2_, and GO *via* an ultrasonication process.^76^ First, GO was dissolved in DI water and sonicated for one hour in order to obtain the NiO/MnO_2_/GO composite (Fig. 7). After adding appropriate quantities of NiO and MnO_2_ to the obtained solution, it was ultrasonically agitated for two hours at room temperature. The resulting composites demonstrated desirable electrochemical characteristics for an asymmetric supercapacitor with excellent energy storage capacity.^76^

**Fig. 7:**
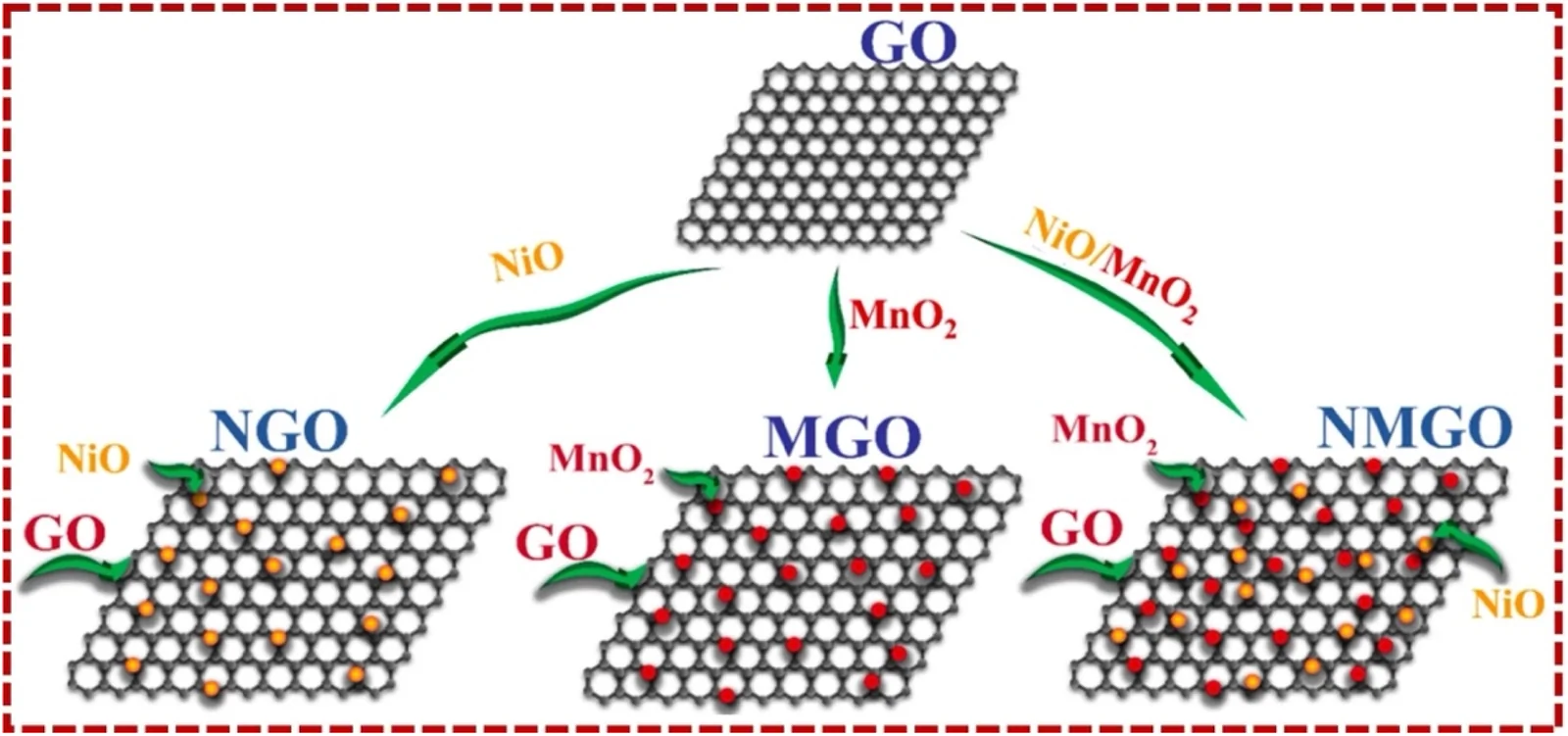
Schematic of the formation of the NiO/MnO_2_/GO composites by the ultrasonication process.^76^ Here, NGO, MGO, and NMGO represent NiO-GO, MnO_2_-GO, and NiO/MnO_2_-GO, respectively. Copyright 2023, MDPI.

The large-scale synthesis of fluorine-doped graphene oxide with improved power density and energy density was achieved through a plasma treatment process for high-performance supercapacitor applications.^33^ The authors mentioned that the inherent covalent bond nature of 2D carbon-based materials may not be suitable for supercapacitor applications. Therefore, finding effective ways to enhance the supercapacitor performance by modifying the covalency of bonding between the graphene-based electrode and K^+^ is necessary. It is noteworthy to mention that KMnO_4_, H_2_SO_4_, and sodium nitrate were used in the synthesis process, and it is a solvent-free scale-up doping approach to obtain FGO through plasma treatment at ambient pressure.^33^

## 3 Morphology of GO-based nanocomposites

The morphology of graphene oxide (GO)-based composites is a primary determinant of charge storage, electron transport, and cycling durability. Structural features should therefore be interpreted not simply as geometric descriptors, but also as the physical origins of measurable electrochemical behavior. Morphology itself depends on the surface chemistry of GO, which governs how individual sheets disperse, associate, and assemble within a composite. The density and spatial distribution of oxygen-containing functional groups regulate sheet aggregation, folding, and packing, as demonstrated by Jiang *et al.*, and their study correlated the GO surface chemistry with the aggregation behavior of both flat and crumpled sheets.^77^ This correlation between surface chemistry and morphology extends directly to electrochemical transport. In reduced GO, the pore structure and residual surface functionality synergistically regulate charge-transfer resistance in aqueous media, with a higher concentration of surface functional groups associated with lower interfacial resistance.^78^ This relationship is particularly important for supercapacitor electrodes because uncontrolled aggregation promotes face-to-face restacking, suppresses ion-accessible surface area, and limits capacitance before an additional electroactive phase is introduced. A structure–performance analysis must therefore begin with the surface chemistry that establishes the morphology.

Electrochemically accessible surface area is the first major structural determinant of capacitance and rate capability. Open, porous, and interconnected architectures expose a larger fraction of electroactive sites while shortening the ion-transport pathways, thereby increasing the specific capacitance and improving its retention at high current density. Dzikunu *et al.* demonstrated this relationship with GO derived from graphitic spent pot lining, where the accessibility and connectivity of the processed carbon surface governed its performance as a supercapacitor electrode.^79^ A similar structure–function relationship was observed for honeycomb-structured graphene produced from acidic GO by chemical expansion, in which an open, interconnected framework increased the accessible surface and improved electrochemical performance.^80^ Evidence from related energy-storage systems further supports this principle. Esteve-Adell *et al.* compared three graphene nanoplatelets and found that increasing the specific surface area from 296 to 714 m^2^ g^−1^ increased the attainable capacity in lithium-ion batteries.^81^ Although these data are not acquired for supercapacitors, they reinforce the broader conclusion that nominal surface area is beneficial only when the electrode architecture keeps that surface electrochemically accessible. Connected porosity, rather than surface area alone, is therefore the more meaningful morphological descriptor of a high-performance electrode.

The morphology also governs interfacial charge transfer by determining the quality and continuity of contact between the electroactive phase and the conductive GO-derived framework. Uniform dispersion of an active material over a percolating conductive scaffold increases the number of electronically coupled interfaces, shortens the electron-transport distances, and consequently, lowers the charge-transfer resistance. Anitha *et al.* illustrated this effect using the morphology-controlled TiO_2_ on reduced GO, where the modification of the oxide nanostructure through a combined anodization and hydrothermal route produced distinct electrochemical responses despite closely related chemical compositions.^82^ A similar influence of morphology is evident in SnSbAg_0.1_ composites, for which the incorporation of reduced GO altered the organization of the active phase and produced corresponding changes in electrochemical behaviour.^83^ These observations establish a general design principle. A finely dispersed active phase with intimate contact to a continuous conductive network should exhibit low interfacial resistance, whereas particle aggregation, thick, poorly conducting domains, or incomplete interfacial contact will increase the resistance even when the constituent materials are intrinsically electroactive.

The mechanical consequences of morphology become increasingly important during prolonged cycling, particularly when the active phase undergoes substantial redox-induced volume changes. Repeated expansion and contraction can generate local stress, particle fracture, interfacial separation, and progressive loss of electrical contact, ultimately causing capacitance decay. A compliant, interconnected GO-derived scaffold can mitigate these processes by dispersing the active phase, anchoring individual domains, and accommodating local strain. This stabilizing role is evident in reduced-GO-modified SnSbAg_0.1_, where the carbon framework improved the structural integrity and electrochemical retention relative to the unsupported active material.^83^ In addition to charge storage, a related structural principle has been demonstrated by Moriau *et al.*, showing that the morphology of high-surface-area supports controls the activity and stability of supported Ir nanoparticles during the oxygen evolution reaction.^84^ Although this example considers electrocatalysis rather than capacitive storage, it provides a useful structural analogy. The architecture of the support determines whether the nanoscale active domains remain accessible, mechanically stabilized, and electronically connected during operation. For active materials that undergo large volume changes, a well-dispersed, anchored, or partially confined morphology should therefore promote long-term stability, whereas unconfined particle growth is expected to accelerate structural degradation.

Overall, these relationships define a predictive morphological framework for GO-based composite electrodes. The most favourable architecture is hierarchical, porous, and interconnected, with sufficient open volume to maintain electrolyte access, continuous conductive pathways to minimize charge-transfer resistance, and mechanically compliant interfaces that accommodate cyclic strain. The composites approaching this structural regime should combine high ion-accessible surface area with intimate contact between GO and the electroactive phase, thereby sustaining rapid charge transport and structural integrity over extended cycling. In contrast, the restacking of GO sheets, aggregation of the active phase, and uncontrolled particle growth simultaneously reduce accessible surface area, disrupt conductive pathways, and concentrate mechanical stress. Such morphologies should therefore exhibit higher interfacial resistance, weaker rate performance, and more rapid capacitance decay.

On the other hand, the distinction between the parent forms of the carbon phase, pristine graphene, GO, and rGO, lies in the trade-off between electrical conductivity, surface functionality, and structural stability, which determines the framework structure. Pristine graphene offers the highest intrinsic conductivity and a theoretical surface area on the order of 2600 m^2^ g^−1^, which favours rapid electron transport and low charge-transfer resistance, yet its inert, functional-group-free basal plane is strongly hydrophobic and prone to irreversible π–π restacking, and hence, it suffers from aggregation in practical electrodes and poor wettability in aqueous electrolytes. GO shows a stark contrast. Extensive epoxy, hydroxyl, and carboxyl groups render it hydrophilic and readily dispersible, which suppresses restacking and favors faradaic pseudocapacitive contribution through redox-active oxygen groups, resulting in the disruption of the conjugated sp^2^ network, which makes GO nearly insulating, and hence, its rate capability and specific capacitance remain limited. Reduced GO represents deliberate compromise between these limits. Controlled removal of oxygen restores conjugation and conductivity, while a residual population of oxygen groups preserve wettability and interlayer spacing, and the resulting balance enables rGO and its three-dimensional assemblies to consistently outperform both pristine graphene, which restacks, and unreduced GO, which cannot conduct. The degree of reduction is the tuning parameter. Mild reduction retains functionality and dispersibility at the cost of conductivity, whereas aggressive reduction maximizes conductivity but drives the material back toward graphene-like hydrophobicity and restacking. Stability follows the same trend. GO is chemically metastable and can degrade or lose oxygen functionality over time, and under bias, pristine graphene is chemically stable but mechanically prone to aggregation that reduces its performance during cycling, and a well-structured rGO framework offers the most durable combination by resisting both chemical degradation and physical collapse. The morphology can thus be designed predictably to integrate ion accessibility, electron transport, and mechanical stability. Such morphological optimization depends on the choice of material, whether graphene, GO, or rGO, providing a direct structural basis for understanding and optimizing the performance of GO-based electrochemical energy-storage materials.

An example of rGO-based nanocomposites (rGO-Au@PANI) was also discussed in previous sections along with its synthesis approach and morphological characteristics. The morphological features of the GO-based nanocomposites are unique and significant for their electrochemical properties. For example, Min Chung *et al.* prepared Ni/GO nanocomposites with a distinctive and tunable morphology in a facile way, which are desirable for supercapacitor applications.^85^ The authors prepared two different Ni/GO composites, namely, Ni/GO-1 and Ni/GO-2 with different nickel contents (3 wt% NiO/GO (Ni/GO-1) and 8 wt% NiO/GO (Ni/GO-2)). For the preparation of Ni/GO-1 and Ni/GO-2, 30 mg and 90 mg, respectively, of Ni(acac)_2_ were added, to 200 mg of GO. These additions influence the morphological properties of the Ni/GO nanocomposites.

Fig. 8 displays the TEM images of the (a–c) Ni/GO-1 and (d–f) Ni/GO-2 nanocomposites and the NiO nanoparticle size distribution of (g) Ni/GO-1 and (h and i) Ni/GO-2. It is interesting to note that the size of the NiO particles was found to be extremely consistent and evenly dispersed on the GO surface, irrespective of the metal loadings. The particle size of Ni was around 20.5 nm, exhibiting a narrow particle size distribution and uniform dispersion at low Ni loadings (3 wt%). A distinctive sea-island-like structure was identified with high Ni loadings (8 wt%). This morphology exhibited two distinct sizes of Ni particles with uniform size distribution: (i) smaller nanoparticles of ∼2.9 nm and (ii) larger nanoparticles of ∼25.5 nm. The larger nanoparticles were found to have an irregular shape, whereas the smaller ones were spherical in shape. From the magnified TEM images, it was observed that several large NiO nanoparticles had a distinctive hexagonal structure (Fig. 8f). Furthermore, the TEM images demonstrated that GO sheets are not agglomerated and have mostly preserved their single-layer structure.^85^

**Fig. 8:**
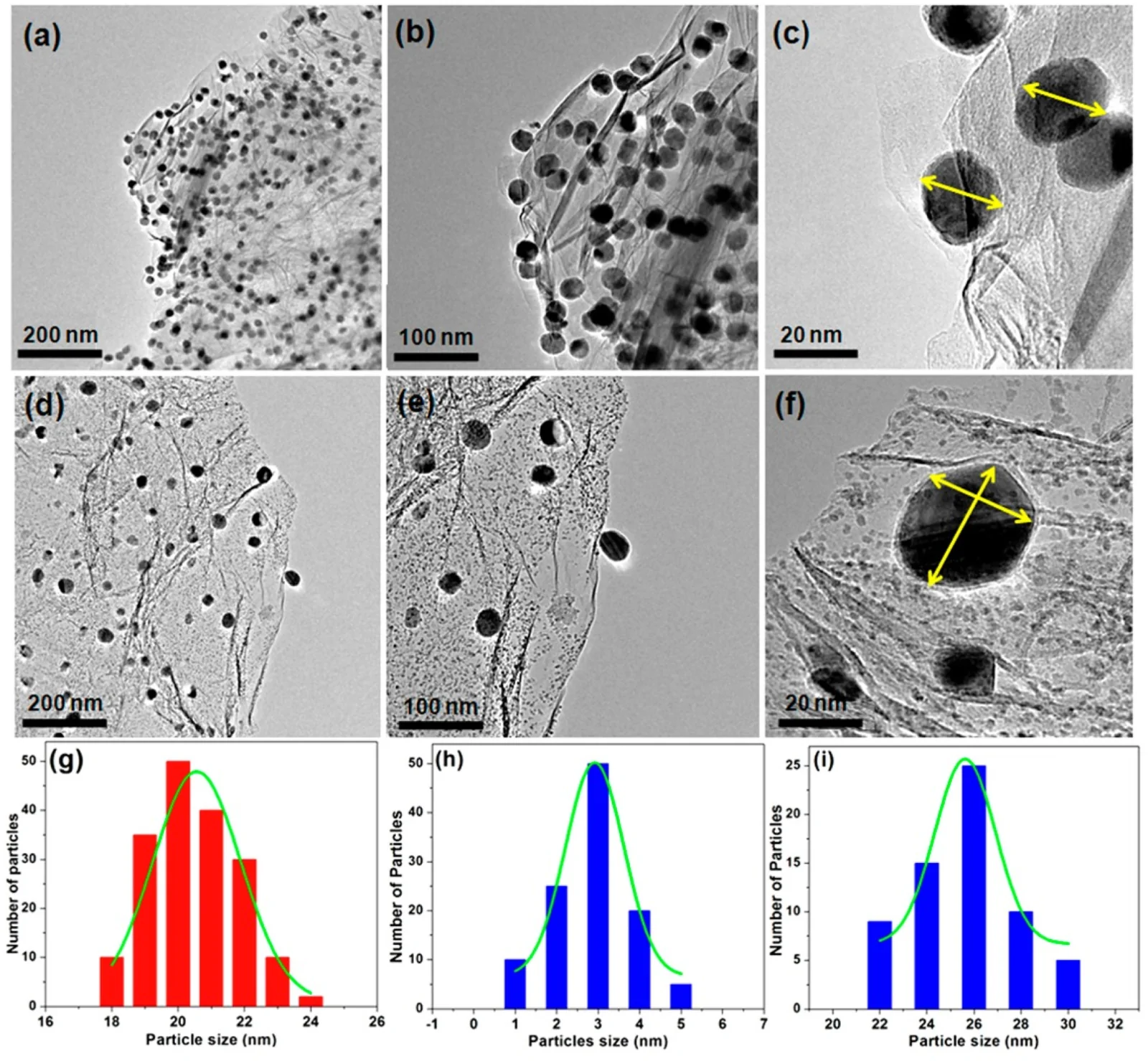
(a–c) TEM micrographs of the Ni/GO-1 and (d–f) Ni/GO-2 nanocomposites. Particle-size distributions of NiO in (g) Ni/GO-1 and (h and i) Ni/GO-2.^85^ Copyright 2019, MDPI.

E. Manikandan *et al.* prepared MnCoS (MCS) and rGO/MnCoS (rMCS) composites with a large surface area, providing many electroactive reaction sites suitable for electrochemical reactions.^59^ Such properties endow the prepared composites with enhanced electrochemical performance. Fig. 9a displays the TEM image of the synthesized rGO/MCS NPs, which are significantly denser and demonstrate improved distribution. The uniform distribution of the nanoparticles leads to an increase in electron transport. Moreover, the well-ordered morphology can provide increased active sites for electrochemical processes, thereby enhancing the total specific capacitance and charge/discharge rates. The HR-TEM image confirms that the lattice rings measure around 0.497 nm and 0.361 nm, which correspond to the (220) and (002) crystal planes of MCS fully developed on rGO (Fig. 9b). Fig. 9c confirms the polycrystalline nature of MCS, displaying brilliant fringes corresponding to the (311) and (220) planes. The homogeneous partition of Mn, Co, S, and C elements is demonstrated in Fig. 9d.

**Fig. 9:**
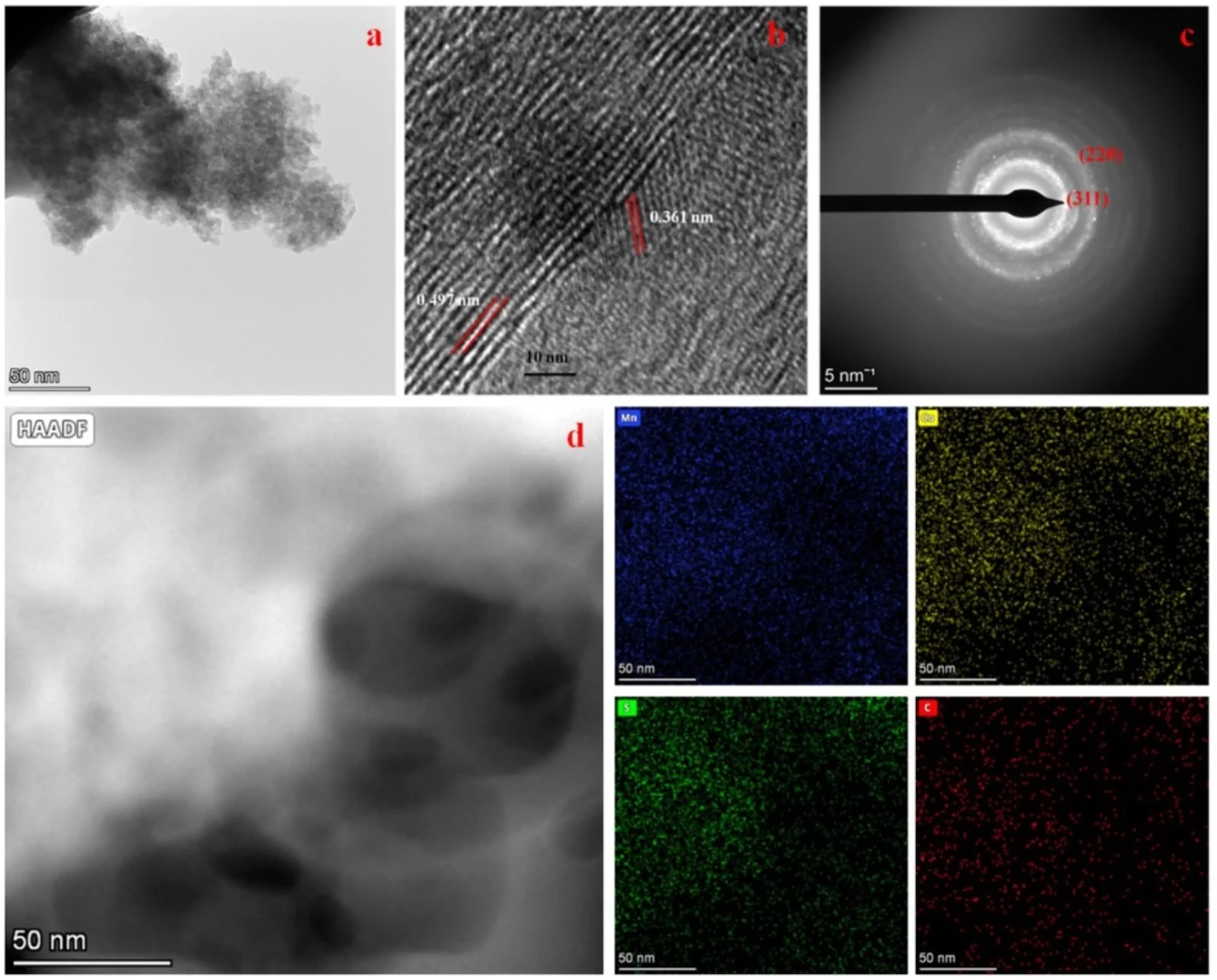
(a) TEM image, (b) HRTEM image, (c) selected-area electron diffraction (SAED) pattern, and (d) STEM image and corresponding elemental mappings of rMCS NPs. Reproduced with permission from ref. 59 Copyright 2024, the author(s).

Rani *et al.* synthesized GO-based NaCr_2_O_4_/GO composites in the nanostructure form with distinct morphological features and superior specific capacitance for energy storage applications.^86^ The authors showed that the nanoparticles exhibit a homogeneous morphology and a quasi-spherical structure. The NaCr_2_O_4_ nanoparticles were bound to the GO nanosheets in the NaCr_2_O_4_/GO nanocomposites, and it is also possible to identify GO nanolayers easily.^87,88^ The identified atomic percentages of Na and Cr in the nanocomposites were 07.07% and 52.53%, respectively, indicating their incorporation in GO and the formation of NaCr_2_O_4_/GO nanocomposites.^86^

Dusan Losic *et al.* prepared rGO/MnFe_2_O_4_, rGO/NiFe_2_O_4_, and rGO/MnFe_2_O_4_/Ppy composites to achieve high gravimetric and areal capacitances for supercapacitors.^89^ For example, the morphologies of GO, rGO/MnFe_2_O_4_, and rGO/MnFe_2_O_4_/Ppy composites are presented in Fig. 10(a–c). The sponge-like porous morphology of the rGO/MnFe_2_O_4_ composites is visible, where the nanorod structure of MnFe_2_O_4_ is distributed on graphene sheets (Fig. 10b). The PPy structure can also be observed in the rGO/MnFe_2_O_4_/Ppy composites (Fig. 10b). This type of morphology facilitates supercapacitor applications utilizing these composite materials.

**Fig. 10:**
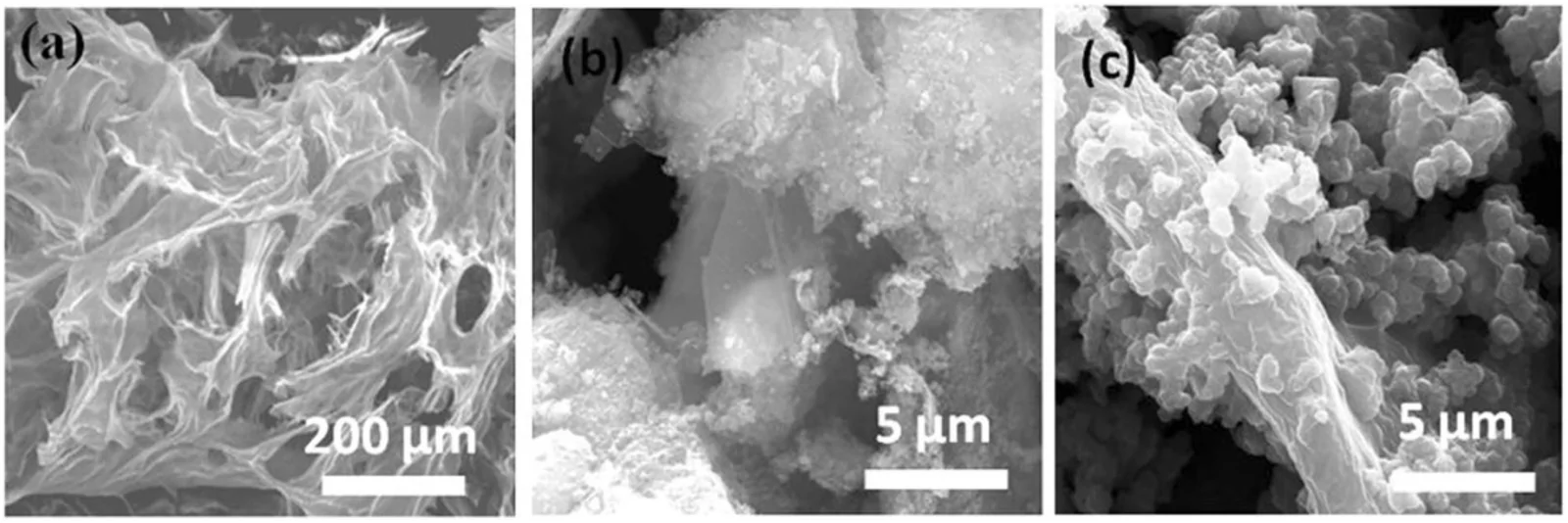
FESEM micrographs of (a) GO (b) rGO/MnFe_2_O_4_, and (c) rGO/MnFe_2_O_4_/Ppy composites. Reproduced with permission from ref. 89 Copyright 2019, the author(s).

Zhao *et al.* synthesized GO/PANI/metal hydroxide composites with controllable morphological features for supercapacitor applications.^90^ The authors reported the honeycomb morphology of GO/PANI/metal hydroxide with the loadings of different contents of nickel ions (Ni^2+^) or cobalt ions (Co^2+^).^90^ Interestingly, the structure of the GO/PANI/metal hydroxide was modified to varying degrees following the progressive doping of Co^2+^. The specific surface area of the GO/PANI/metal hydroxide nanocomposites decreased, and the transmission capability of electrolyte ions was impaired, which adopted a cellular concave shape when doped alone with Co^2+^. In this case, the PANI nanorods are uniformly dispersed on the intact graphene sheet surface with Ni^2+^ doping. Hence, the regular layer of PANI on graphene (GO/PANI/Ni^2+^) is able to optimize its electrochemical performance, ionic diffusion, and electrical conduction.^90^ Thus, the appropriate structure of GO-based nanocomposites is an important factor in achieving high-performance supercapacitors. In most cases, the agglomerated and inhomogeneous morphology of the prepared nanocomposites limited their performance for supercapacitors. These characteristics also limit their large-scale applications in the areas of energy storage, like supercapacitors. Thus, more attention must be paid to achieve a suitable morphology of GO-based nanocomposites for effective use in device fabrication for supercapacitors.

## 4 Supercapacitor applications

The electrochemical parameters are the primary factors for an HPS. Therefore, it is desirable to design and prepare suitable GO-based nanocomposites as electrodes for energy storage devices like supercapacitors. It is also noteworthy that an ideal electrode material is required for enhancing the electrochemical properties of HPSs. The vital features of an ideal electrode for supercapacitors are controllable morphology with a large surface area, thermal stability, and comparatively low production cost.^91^ Therefore, scientists are searching for desirable materials to improve the features of electrodes for achieving the superior performance of supercapacitors. In this context, GO-based nanocomposites can be used as electrode materials by modifying their electrochemical properties. Various GO-based composites and their electrochemical properties, including electrode configuration, are presented in Table 2. In the following subsection, the potential of different GO-based nanocomposites for use as electrode materials is discussed.

**Table 2:** GO-based composites and their electrochemical properties^a^

Composites	Electrode configuration	Specific capacitance (F g^−1^)	Energy density (Wh kg ^−1^)	Power density (W kg^−1^)	Ref.
RuO_2_/rGO	Three	270	15 W h kg^−1^	76.4 k W kg^−1^	92
rGO-MnO_*x*_	Three	398.8	23.3 Wh kg^−1^	2001 W kg^−1^	93
rGO-Mn_3_O_4_	Three	248.1	17.0 Wh kg^−1^	1997.6 W kg^−1^	93
GO-nickel sulfide nanoplates	Three	1745.50	87.30	1010	94
GO@nickel cobalt phosphide	Three	1125	27.71	788 W kg^−1^	95
rGO-CuS	Three	109	44	1.4 kW kg^−1^	96
ZnS/rGO/PANI	Three	722	349.7	18 kW kg^−1^	97
Nickel–cobalt phosphide/multilayer graphene (NiCoP)/(MLG)	Three	1419.6	32.19	741.65 W kg^−1^	98
rGO/PTh/CB	Two	930.62	42.47	1532	99
rGO/Ag/PTh	Two	953.13	28.8	2843.3	100
GO/PTh	Three	296	148	41.6	101
PDMOT/rGO/SnS_2_	Three	1182	171	2457	102
PEDOT/rGO/HfS_2_	Three	1015	171	2457	103

a Abbreviations: CB = carbon black, PDMOT = poly(3,4-dimethoxythiophene), and PEDOT = poly[3,4-ethylenedioxythiophene].

### 4.1. GO and metal oxide nanocomposites

Numerous researchers combined metal oxides (MOs) with GO to prepare nanocomposites with enhanced electrochemical properties for HPSs. In this regard, ruthenium oxide (RuO_2_)/rGO composites were synthesized at different pH levels without subsequent calcination.^92^ The authors investigated the supercapacitor performance of the prepared RuO_2_/rGO composites in 1.0 M NaNO_3_ in a three-electrode system. The RuO_2_/rGO composites fabricated at pH 8.0 showed the highest specific capacitance of 270 F g^−1^ (238.5 mF cm^2^) and energy and power densities of 15 W h kg^−1^ and 76.4 k W kg^−1^, respectively. At 24 A g^−1^, the suggested supercapacitor shows exceptional cycling stability over 5000 cycles and a steady potential window up to 1.1 V.^92^ However, Ru^3+^ ion reduction was not possible during the synthesis process at pH 2.0, and as a result, the capacitive behavior was poor. rGO-MnO_*x*_ and rGO-Mn_3_O_4_ composites were also prepared by Singu *et al.*,^93^ and electrochemical capacitive responsive properties of both the composites were compared. The author demonstrated that the rGO-MnO_*x*_ composite has superior electrochemical capacitive responsiveness compared to rGO-Mn_3_O_4_. They also showed that the composites had almost symmetrical anode and cathode curves, suggesting that the nanocomposite electrodes exhibit good reversibility and nearly perfect capacitive behavior. The shape of the CV curves remained rectangular as the voltage sweep rate improved from 5 to 100 mV s^−1^, indicating that the electrodes made of rGO-MnO_*x*_ and rGO-Mn_3_O_4_ nanocomposite materials have outstanding rate capacity throughout a broad range of sweep rates. At a sweep rate of 5 mV s^−1^, the rGO-MnO_*x*_ composite shows a maximum capacitance of 398.8 F g^−1^.^93^ The GCD profile of the rGO-MnO_*x*_ and rGO-Mn_3_O_4_ nanocomposites was found to be symmetric, which indicates the swift charge–discharge behaviour. The capacitance retention of rGO-MnO_*x*_ and rGO-Mn_3_O_4_ was about 80% and 71%, respectively, after 5000 charge–discharge cycles. Due to the strong synergy between MnO_*x*_ and rGO, as well as the effective prevention of graphene nanosheet stacking during electrochemical cycling, rGO-MnO_*x*_ exhibits exceptional cycling performance. The highest energy density and power density of the rGO-MnO_*x*_ nanocomposite are 23.3 Wh kg^−1^ and 2001 W kg^−1^, respectively. These values are much higher than those of rGO-Mn_3_O_4_ (17.0 Wh kg^−1^ and 1997.6 W kg^−1^). This is due to the combined effect of rGO and manganese oxide nanoparticles, which have spherical and cubical shapes and a high surface area.^93^

Dusan Losic *et al.* fabricated supercapacitors utilizing rGO/MnFe_2_O_4_, rGO/NiFe_2_O_4_, and rGO/MnFe_2_O_4_/Ppy composite materials.^89^ The electrochemical properties of the prepared materials are displayed in Fig. 11(a–g). From Fig. 11a, it is obvious that the rGO/MnFe_2_O_4_/Ppy CV curve area is larger than the rGO/MnFe_2_O_4_ curve area, indicating that the former has superior capacitance over the latter. Similar outcomes were also displayed for rGO/NiFe_2_O_4_ and rGO/NiFe_2_O_4_/Ppy, as reported by the authors.^89^

**Fig. 11:**
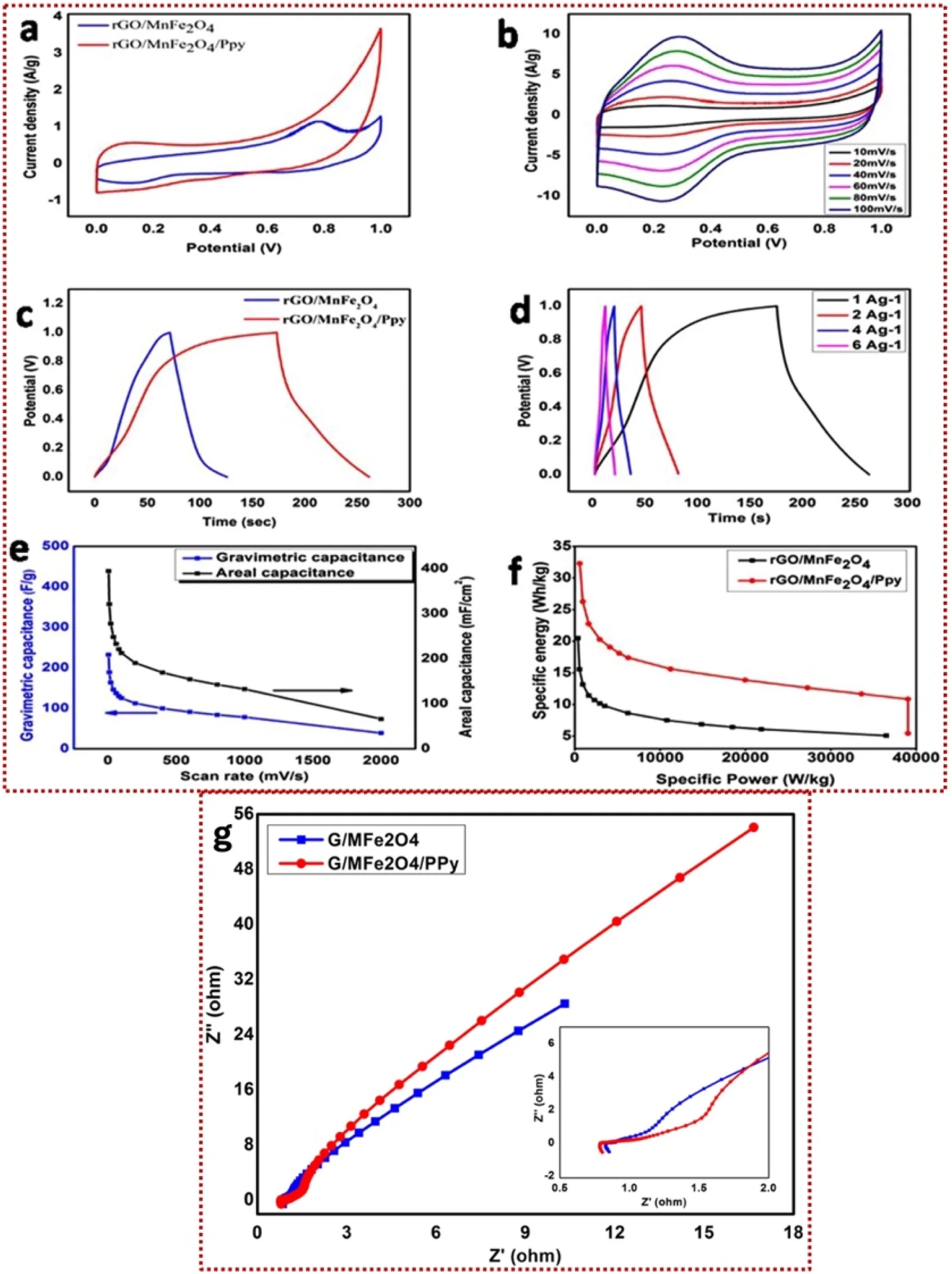
(a) CV profiles of rGO/MnFe_2_O_4_ and rGO/MnFe_2_O_4_/Ppy at 5 mV s^−1^, (b) CV profiles of rGO/MnFe_2_O_4_/Ppy at different scan rates, (c) GCD profiles of rGO/MnFe_2_O_4_ and rGO/MnFe_2_O_4_/Ppy, (d) GCD profiles of rGO/MnFe_2_O_4_/Ppy at different current densities, (e) gravimetric and areal capacitance of rGO/MnFe_2_O_4_/Ppy at different scan rates, (f) Ragone plots and (g) Nyquist plots of the rGO/MnFe_2_O_4_ and rGO/MnFe_2_O_4_/Ppy composites. An enlarged view of the low-impedance region for rGO/MnFe_2_O_4_ and rGO/MnFe_2_O_4_/PPy composites is presented in the inset. Reproduced with permission from ref. 89. Copyright 2019, the author(s).

Amongst all electrodes, rGO/MnFe_2_O_4_/Ppy exhibits the highest current density at the identical scan rate. It has the best capacitance performance compared to all the fabricated electrodes and is a good material for supercapacitor electrodes because of its high current density and corrected behavior in both anodic and cathodic CV profiles. At a scan rate of 5 mV s^−1^, the rGO/MnFe_2_O_4_/Ppy electrode's gravimetric capacitance was 232 F g^−1^ and its areal capacitance was 395 mF cm^−2^.^89^ Because of the larger synergistic impact between the composite components, it is evident that the gravimetric and areal capacitance of rGO/MnFe_2_O_4_/Ppy was 1.57 times higher than that of rGO/MnFe_2_O_4_. The current density increases with the increasing scan rate, according to the reported data, which indicates that the electrode material exhibits higher conductivity, lower internal resistance, and good rate capacity. The GCD curves of rGO/MnFe_2_O_4_ and rGO/MnFe_2_O_4_/Ppy shown in Fig. 11c demonstrate results that are compatible with CV data. The discharge duration of rGO/MnFe_2_O_4_/Ppy is significantly longer than that of rGO/MnFe_2_O_4_. The nearly identical shape of all GCD curves at various current densities presented in Fig. 11d indicates that the electrode material exhibits optimal capacitive behavior.^89^ The gravimetric capacitance and areal capacitance of rGO/MnFe_2_O_4_ and rGO/MnFe_2_O_4_/Ppy were measured using CV and charge–discharge analyses at scan rates of 5–2000 mV s^−1^.^89^ The high electrical conductivity of Ppy, which enhances ion diffusion into the electroactive electrode material, coupled with the synergistic effect of the individual components, explains the superior electrochemical performance of the material observed upon its addition.^89^ The authors reported specific power values of 368.5 W kg^−1^ and 581 W kg^−1^ at 5 mV s^−1^ for rGO/MnFe_2_O_4_ and rGO/MnFe_2_O_4_/Ppy, respectively. At 2000 mV s^−1^, the values increased to 36 479.5 W kg^−1^ and 39 034 W kg^−1^.^89^ The addition of Ppy to rGO/MnFe_2_O_4_/Ppy results in improved electrical conductivity as well as specific energy. The EIS data acquired at an excited potential of 5 mV over a frequency range of 0.01–100 kHz are displayed in Fig. 11g. The Nyquist figure shows that at high frequencies, rGO/MnFe_2_O_4_ and rGO/MnFe_2_O_4_/Ppy exhibit very tiny semicircles.^89^ Thus, all these findings demonstrate that it is feasible to create synergistic composite electrode materials by combining graphene and metal-doped iron oxide CPs. This could be an encouraging approach for modifying the supercapacitor characteristics for achieving extraordinary performance.

The rGO-cobalt oxide (rGO@Co_3_O_4_/CoO) hybrids were prepared by Kumar *et al.*^104^ for advanced supercapacitor electrodes. The prepared hybrids exhibited a specific capacitance of 276.1 F g^−1^ at a scan rate of 5 mV s^−1^ and long-term cycling stability with 82.37% capacitance retention using 0.1 M KOH. The nanoparticle form of Co_3_O_4_/CoO with conducting scaffolds of rGO will offer a promising substitute for electrode materials and show promising applications in supercapacitors.^104^ Gryglewicz *et al.*^105^ synthesized PPy/Fe_2_O_3_/rGO composites, achieving a notable capacitance retention rate. A greater contribution of pseudocapacitive PPy to total capacitance is indicated by the generated nanocomposite material's specific capacitance of 140 F g^−1^ at 0.2 A g^−1^, which is 70% greater than that of the iron oxide/rGO binary composite. During 5000 cycles at 1 A g^−1^, the produced composite shows a remarkable 93% capacitance retention rate. These outcomes are owing to the synergistic effect between PPy and Fe_2_O_3_/rGO composites.^105^ Another example of the easy fabrication of Fe_3_O_4_/rGO nanocomposites with higher capacitance is based on their nanorod structure.^106^ Such behavior was attributed to the small size of prepared nanocomposites, which facilitates the efficient transport of electrolyte ions. The composite exhibited specific capacitances of 504, 138, and 193 F g^−1^ for distinctive materials like Fe_2_O_3_ nanorods/rGO, Fe_2_O_3_ nanoparticles/rGO, and Fe_2_O_3_ assembled nanorods/rGO, respectively.^106^

Rani *et al.* prepared multinary-type GO@SrO@CoCrO_3_@FeCr_2_O_4_@SnO_2_@SiO_2_ nanocomposites to achieve notable electrochemical performance.^107^ The prepared ternary composites exhibited an electron transfer rate of 4.88 × 10^−9^ cm s^−1^ with a capacitance value of 7182 F g^−1^ at a scan rate of 10 mV s^−1^. The GCD analysis showed a power density of 3591.18 to 2163 W kg^−1^ and an energy density in the range of 299 to 120 Wh kg^−1^.^105^ Consistent with these results, a novel multinary nanocomposite shows promise for application as an electrode. Metal oxides like MnCo_2_O_4_ were combined with graphene to fabricate MnCo_2_O_4_/3D-graphene for supercapacitors with improved electrochemical behaviors.^108^ The resulting composite materials exhibit superior cycling stability and a notable specific capacitance of 503 F g^−1^ at 1 A g^−1^ current density. At a current density of 10 A g^−1^, 97.4% of the specific capacitance was maintained after 5000 charge/discharge cycles.^108^ These results indicate the good ability of these composites for use in supercapacitors.

### 4.2. GO and metal sulfide nanocomposites

Metal sulphide nanocomposites based on GO have various advantages in supercapacitor applications, such as higher stability, improved electrical conductivity, and enhanced specific capacitance. GO is a suitable electrode material owing to its large surface area, which, combined with the pseudocapacitive behavior of metal sulfides, results in high energy and power densities. Using nickel sulphide and GO thin films on nanofluids, composites of GO-nickel sulphide nanoplates (GO-NSNPs) were prepared by Razaq *et al.*^94^ In this case, the GO layer helped to significantly improve the electrical conductivity and the surface area of the prepared composites. In CV curves, GO-NSNPs displayed oxidation and reduction peaks, suggesting a pseudocapacitive nature. The GCD profiles, which were performed using a three-electrode setup, showed specific capacitance and specific energy of 1745.50 F g^−1^ and 87.30 Wh kg^−1^ at 5 mA cm^−2^, respectively. A symmetric measurement with two electrodes yielded a specific energy of 15.72 Wh kg^−1^ at 1 mA cm^−2^. Thus, to achieve good performance, GO-NSNPs electrodes can be used in both symmetric and asymmetric supercapacitor applications.^94^

Copper tin sulfide (Cu_4_SnS_4_ (CTS))-rGO/nickel foam (NF) composites with outstanding electrical conductivity were successfully synthesized as electrode materials for supercapacitors.^109^ The authors reported improved supercapacitor performance of the CTS-rGO/NF electrode compared to CTS/NF. At a current density of 5 mA cm^−2^, the CTS-rGO/NF composite electrode demonstrated a dramatically enhanced specific capacitance of 820.83 Fg^−1^, higher than that of CTS/NF.^109^ Additionally, the fabricated composite demonstrated exceptional performance as an electrode for energy storage functions, including SCs, with a better rate capability of 73.1% and superior cycling stability with over 92% capacity retention after 1000 constant GCD cycles.^109^ A comparative CV and GCD profile of CTS−rGO/NF and CTS/NF electrodes is displayed in Fig. 12a and b. Further, the CV and GCD profiles of the CTS-rGO/NF electrode evaluated in 1 M KOH are given in Fig. 12c and d. The higher specific capacitance of CTS-rGO/NF than that of CTS/NF is demonstrated in Fig. 12e. This performance may be related to the enlarged surface area and improved conductivity attributed to the presence of rGO in the composite sample.^109^ The worthy cycling stability of the CTS-rGO/NF electrode and its appropriateness for SC application were confirmed by the much better capacity retention of CTS-rGO/NF electrodes than that of CTS/NF (Fig. 12f). Further, the Nyquist plots of CTS/NF and CTS-rGO/NF and the Bode plot displaying the phase angle for the CTS/NF and CTS-rGO/NF electrodes are displayed in Fig. 12g and h, respectively. These outcomes indicate the superior capacitive characteristic of CTS-rGO/NF. The overall electrochemical performance can be attributed to the synergistic effect of the increased conductivity and surface area resulting from the addition of rGO in the composites.

**Fig. 12:**
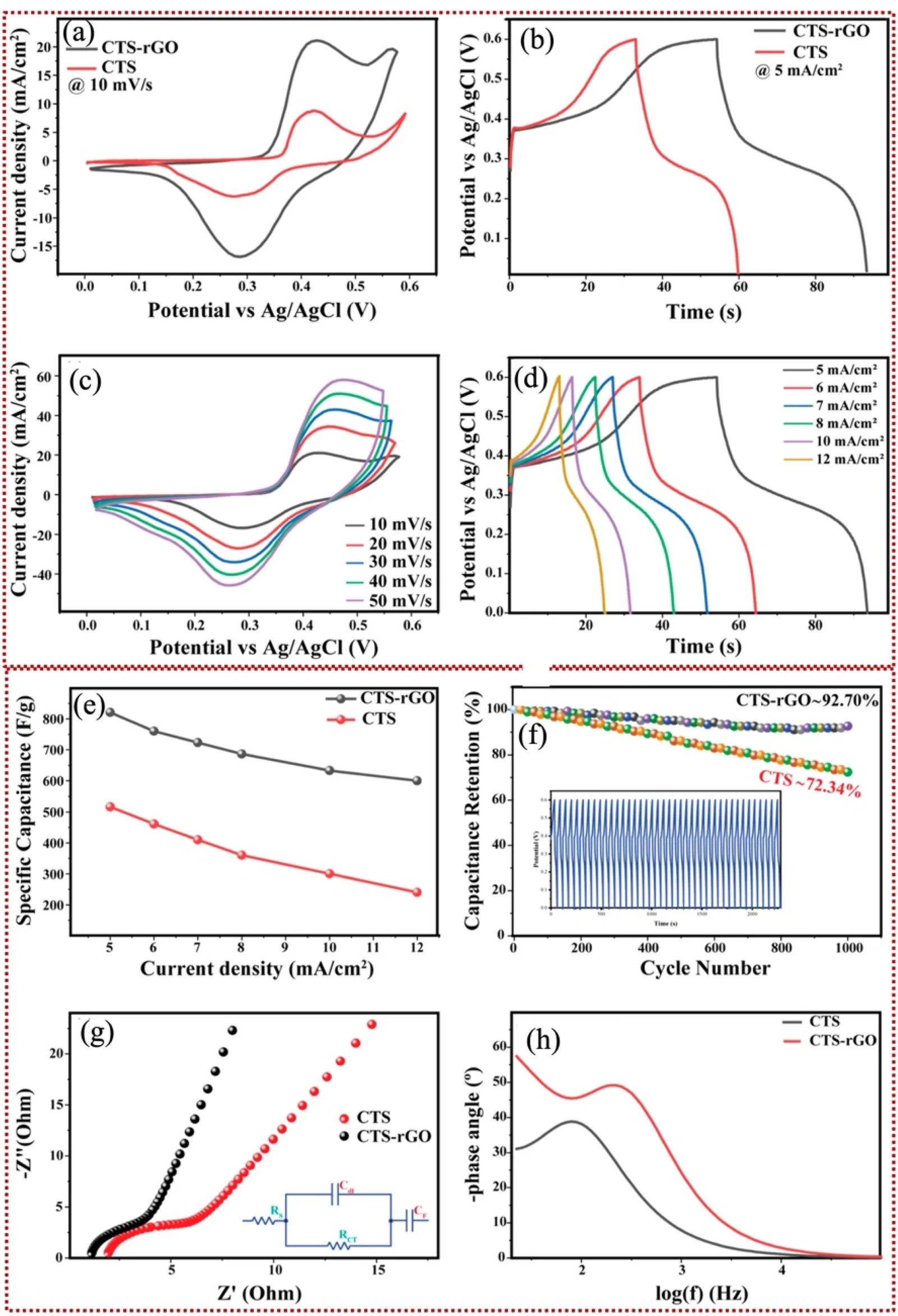
(a) CV profile and (b) GCD profile of the CTS-rGO/NF and CTS/NF electrodes. (c) CV profiles and (d) GCD profiles of the rGO-CTS/NF electrode evaluated in 1 M KOH. (e) Specific capacitance of CTS/NF and CTS-rGO/NF at different current densities. (f) Capacity retention graphs of the CTS/NF and CTS-rGO/NF electrodes showing stability after 1000 GCD cycles at a current density of 7 mA cm^−2^ (g) Nyquist plots of CTS/NF and CTS-rGO/NF. The inset shows the equivalent electrical circuit used to model the electrochemical impedance spectra of CTS/NF and CTS-rGO/NF electrodes; (h) Bode plot displaying the phase angle for CTS/NF and CTS-rGO/NF. Reproduced with permission from ref. 109. Copyright 2024, the author(s).

The SnS/G nanocomposites were prepared by using GO. The electrochemical properties were evaluated by CV, which revealed the pseudocapacitive nature of the composites with the highest specific capacitance of 1398.93 F g^−1^, excellent cycling stability, and a capacitance retention rate of 94.37% for 1000 cycles.^58^ In this work, the prepared composites showed a lesser charge transfer resistance than that of SnS. Such behavior may be attributed to the presence of graphene sheets, which are able to develop an electron route and thus decrease the electrochemical resistance of SnS/G nanocomposites. Remarkably, the composites offer more active sites, which provide high electron conductivity and a large surface area. The composites' exceptional performance suggests that they possess significant electroactive components for use in supercapacitors.

Utilizing rGO and CuS, rGO-CuS nanocomposites were prepared for electrode materials with a specific surface area of 77 m^2^g m^−1^.^96^ In the composites, CuS revealed a hexagonal crystal structure with a mean pore size of 22 nm. At a sweep rate of 5 mV s^−1^ in an aqueous electrolyte of 1 M LiClO_4_, the specific capacitance of the rGO-CuS electrode was measured to be 1201 F g^−1^. The layered structure of the prepared composites facilitates major charge storage through a diffusion-controlled process. A specific capacitance of up to 109 F g^−1^ is achieved by the rGO-CuS-based symmetric supercapacitor using a PVA-LiClO_4_ gel electrolyte at a sweep rate of 5 mV s^−1^. Besides, at an applied current of 5 mA, after 6000 cycles, a specific energy of 44 Wh kg^−1^ and a specific power of 1.4 kW kg^−1^ were achieved with 87% stability. This straightforward formation of a layered hybrid electrode for flexible supercapacitors shows promise for application in wearable electronics and smart textiles. The Ni_3_S_2_/3D graphene/nickel foam composite was reported for good electrochemical supercapacitor applications.^52^ The prepared electrode is stable, demonstrating an outstanding specific capacitance within the limit of 2585 F g^−1^ with a current density of 1 A g^−1^ and a high capacitance retention rate of ∼70.68%. The significantly increase in the conductivity and specific surface area of the as-prepared composite may be due to the ideal Ni_3_S_2_ heazlewoodite phase and suitable contact between Ni_3_S_2_ and 3D G/NF. These findings may be useful for achieving practical supercapacitor applications.^52^ Good electrochemical performance was also achieved using the nanostructure form of graphene-encapsulated NiS/Ni_3_S_4_ materials.^110^ After 5000 cycles, the prepared nanocomposites showed good cycling stability (88%) and coulombic efficiency (95%) at a practically relevant specific current of 70 A g^−1^. It also possesses a high specific capacity of 827C g^−1^ at 5 A g^−1^.^108^ Because of its elevated conductivity, synergistic interaction between graphene and NiS, and mesoporous graphene-encased nanostructure, NiS/Ni_3_S_4_ exhibits exceptional electrochemical performance. At 2 A g^−1^, the asymmetric supercapacitor device based on nanocomposites and rGO has the highest energy density of 86.3 W h kg^−1^. Thus, the nanocomposite is believed to be a prospective electroactive material for supercapacitors owing to its outstanding electrochemical properties.^110^

Zhang *et al.* fabricated NiCo_2_S_4_@rGO composites for electrode applications.^111^ Compared to previous solitary metal oxides or sulphides, the produced electrode had a higher capacitance and showed exceptional electrochemical activity because of the presence of active sites. At a current density of 1 A g^−1^, the synthesized electrode achieved a specific capacitance of 2418 F g^−1^. A wide operating voltage window of 1.6 V can be achieved by using NiCo_2_S_4_@rGO as the cathode and N-doped graphene as the anode in the asymmetric supercapacitor.^111^ This prospective working window enables its applicability in various industries. Further, the prepared materials achieved a high rate capability and excellent cycling stability due to the combined contributions of pseudocapacitance and double-layer capacitance. At a power density of 411 W kg^−1^, the apparatus produced the highest energy density of 34.1 Wh kg^−1^. Furthermore, the device exhibited notable cycling stability, maintaining 86.4% of its capacitance after 5000 cycles. The authors mentioned that the suggested approach may offer a broad path for designing supercapacitors with a large voltage window and high capacitance.^111^

### 4.3. GO and metal nitride nanocomposites

The improved electrochemical performance caused by the synergistic effects of GO and metal nitrides is the main advantage of GO-based metal nitride composites in supercapacitor applications. The redox activity and pseudocapacitance of metal nitrides, together with GO's high surface area and conductivity, result in superior capacitance, rate capability, and stability. In this context, metal nitrides like vanadium nitrides have great promise for use with GO for supercapacitor applications. Compared to noble metal oxides, vanadium nitride has superior electrical conductivity, superior chemical stability, and a higher theoretical capacitance. Therefore, an attempt was made by Yuan *et al.* to prepare composites of VNNWs@rGO utilizing vanadium nitride nanowires (VNNWs) and rGO.^112^

The hierarchical vanadium nitride nanowires (VNNWs)@rGO composite showed exceptional supercapacitor performance, with elevated capacitances of 222 and 65 F g^−1^ 104 at current densities of 0.5 and 10 A g^−1^, respectively.^112^ The boosted electrochemical ability is related to the distinctive structural design: the cavities and nanoparticles inside the VNNWs can offer a good number of active sites for electric double-layer capacitors and pseudocapacitance; N-doped rGO endows superior electric conductivity and chemical stability for vanadium nitride; and the interlinked laminar network of the prepared composites is essential for electrolyte penetration and charge transfer.^112^ Another attempt was made to prepare metal-free mesoporous carbon nitride (MCN)-graphene aerogel (GA) nanocomposites with remarkable supercapacitor performance among the diverse materials of the carbon family.^113^ Compared to the carbon nitride (142 F g^−1^) and graphene aerogel (174 F g^−1^) electrodes, the produced nanocomposite has a specific capacitance of around 240 F g^−1^ at 5 mV s^−1^ in 1.0 M H_2_SO_4_. The generated symmetric supercapacitor has a superior specific energy of 11.6 W h kg^−1^, an exceptional specific power of 8.0 kW kg^−1^, and an extraordinary capacitance retention of >94% after 10 000 charge–discharge cycles, demonstrating good long-term stability. These outcomes indicate the potential of the prepared nanocomposites for future energy storage applications and other related technologies.^113^

The large-scale preparation of boron nitride (h-BN)/graphene hybrid films with heterostructures for flexible supercapacitors was reported in a previous study.^114^ Because of the synergistic effects of h-BN and graphene, the produced film demonstrated excellent volumetric capacitance, remarkable rate capability, long cycling life, and elevated flexibility that are suitable for supercapacitor applications. Fig. 13 demonstrates the electrochemical properties of the h-BN/rGO hybrid film for a flexible supercapacitor. A prototype flexible supercapacitor device was fabricated utilizing the optimized hybrid film (Fig. 13a). Fig. 13b displays the CV profile of the hybrid film-based flexible supercapacitor, which demonstrated steady device operation even after bending and a volumetric capacitance of approximately 95 F cm^−3^ at 50 mV s^−1^. Fig. 13c presents the charge/discharge curve of the flexible device.^114^ Furthermore, despite the significant capacitance changes observed during the continued test, the device showed 100% capacitance retention even after 1000 bending tests (Fig. 13d). This suggested that the hybrid film-based flexible supercapacitor had decent long-term stability and flexibility. Thus, the fabricated films show great promise for applications in electronics and energy storage devices and are used as scalable, flexible electrodes in supercapacitors.

**Fig. 13:**
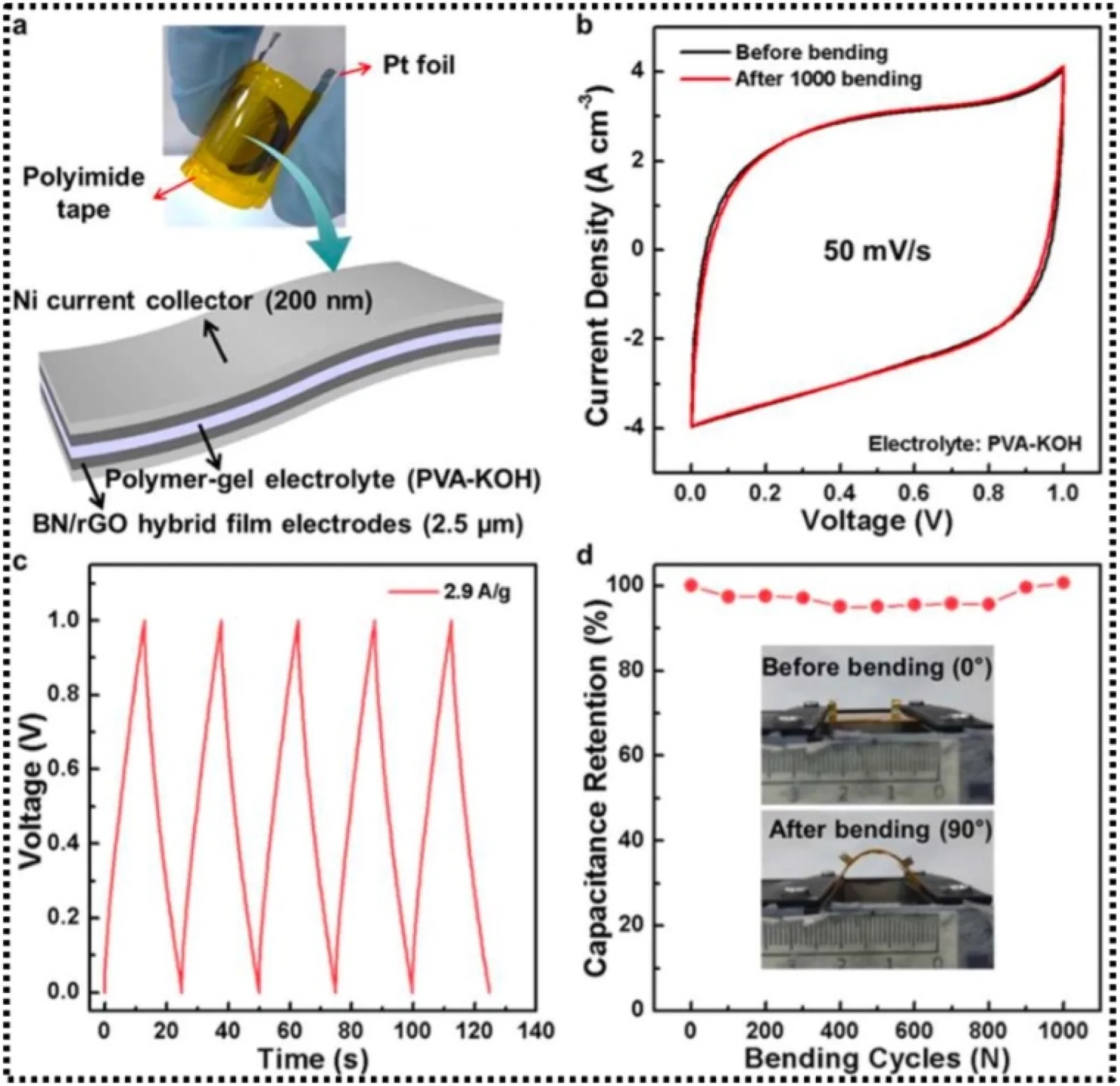
Flexible supercapacitor based on a BN/rGO hybrid film. (a) Schematic of the BN/rGO film-based flexible supercapacitor and a digital image demonstrating the device's flexibility. (b) CV profile of the 0.5 : 4 BN/rGO-based device revealing the same integrated area after 1000 bending cycles. (c) Film's charge/discharge curve with no discernible IR drop at a current density of 2.9 A g^−1^. (d) Mechanical characteristics of the neat NH_2_-rGO and hybrid films show that an optimum concentration of BN in the hybrid film served as a mechanical nanofiller and increased the film's tensile strength. Reproduced with permission from ref. 114 Copyright 2016, the American Chemical Society.

Ran *et al.*^115^ showed a simple technique for creating a composite of vanadium nitride nanoparticles (VNNP)/GO utilizing GO and vanadium nitride nanoparticles. The as-synthesized VNNP/GO composites had a specific capacitance of 109 F g^−1^ at a current density of 1 A g^−1^. Even after 5000 cycles at a current density of 2 A g^−1^, an excellent 93% capacitance retention was achieved. The semicircular diameter of vanadium nitride and GO composites in EIS was higher than that of vanadium nitride; nevertheless, the slope of the curve in the low-frequency area range was lower, indicating a low diffusion resistance. In order to guarantee that the electrode works smoothly in the charging and discharging process, the electrochemically active materials' efficiency is significantly increased by the synergistic impact of vanadium nitride and the good conductive nature of GO.^115^

It is worthwhile to fabricate electrode materials using transition metal nitrides since they are potential materials for supercapacitors because of their great electrochemical capability and superior chemical stability. However, controlling crystallinity and electrochemically active sites in the typical synthesis processes of these materials is still difficult. In this regard, a TiN/vertical graphene (VG) nanohybrid was created for use in HPSs.^116^ The TiN/VG hybrid exhibits an areal capacitance that is more than four times greater than that of the commercial TiN-plated VG hybrid when aqueous Li_2_SO_4_ works at a voltage window of 1.0 V. The TiN/VG can obtain an areal capacitance of 9.0 mF cm^−2^ at a scan rate of 100 mV s^−1^ when the voltage window is enlarged to 1.8 V.^116^ After 10 000 cycles, the electrode retains 89.5% of its initial capacitance, which is one of the highest values documented for TiN nanoparticles.^116^ Swain *et al.*^117^ created rGO/gallium nitride (GaN) nanocomposites. Utilizing CV, the ultimate specific capacitance of 454 F g^−1^ was found at a scan rate of 10 mV s^−1^. The GCD approach yielded similar results, with specific capacitance ranging from 116.8 to 146.1 F g^−1^ at a current density of 1 A g^−1^.^117^ Additionally, an extremely low charge transfer resistance was observed, with an electrochemical impedance spectroscopy analysis maximum of just 2.36 Ω.^117^ Thus, the RGO–GaN nanocomposites exhibited a good capacitive performance, useful for supercapacitor applications.

In the realm of wearable technology, graphene-fiber (GF) supercapacitors have garnered a lot of interest. However, high-energy-density active materials are still required. Compared to traditional transition metal oxides or CPs, transition metal nitrides are promising candidates for this purpose because of their better electrical conductivity, stability, and pertinent electrochemical characteristics. Kim *et al.* created high-performance hybrid fiber (WN-rGOF) supercapacitors by effectively combining tungsten nitride (WN) and rGO fibers.^118^ These hybrid supercapacitors attained an energy density of 1.448 mW h cm^−3^ and an elevated capacitance of 16.29 F cm^−3^ at 0.05 A cm^−3^, which are 1.75 and 7.5 times greater than those of the tungsten oxide/rGO hybrid fiber (WO_3_–rGOF) supercapacitor and pure rGOF supercapacitor, respectively.^118^ When three WN-rGOF supercapacitors were linked in series, the energy density easily increased to 2.896 mW h cm^−3^. The WN-rGOF supercapacitor demonstrated notable performance under extreme mechanical deformation and an elevated capacitance retention of 84.7% after 10 000 cycles.^118^

### 4.4. GO and CP nanocomposites

CP composites based on GO are promising materials for supercapacitor applications because of their improved electrochemical and electrical conductivity, especially when combined with the elevated conductivity and vast surface area of graphene. CPs like PANI, PPy, and poly(3,4-ethylenedioxythiophene) (PEDOT) when combined with graphene or graphene oxide (GO) yielded composites, which showed improved supercapacitor performance by boosting the capacitance, power density, and cycle life. In this regard, Qin *et al.* fabricated graphene/PANI composites, which were applied as the symmetric all-solid-state electrode.^119^ With good rate capability and cycling stability, the electrochemical tests of the resultant rGO/PANI showed a capacitance of 853.7 F g^−1^ at 1 A g^−1^. Furthermore, the symmetric all-solid-state capacitor is fabricated using the hydrogel composite without any additives, achieving a capacitance of 741.8 F g^−1^ with an elevated capacitance retention of 92.6% over 8000 cycles.^119^ These exceptional electrochemical properties of the composite film are attributed to the synergistic effects of PANI's exceptional pseudocapacitive property and the high conductivity and porous structure of the rGO matrix.^119^

An electrode for supercapacitor applications was fabricated by Chen *et al.*^69^ using rGO/PANI/PVA nanocomposites.^120^ The resulting PANI/PVA nanofibers show pseudocapacitance behavior, while rGO/PANI/PVA demonstrates the electrochemical features of the double-layer capacitor. At 0.2% rGO concentration, rGO/PANI/PVA likewise shows a constant 119.8 nm diameter distribution with no adhesion of beads or droplets. Because of its form, the fibers' specific surface area is increased, and enough channels are provided for ion transport. In comparison to the PANI/PVA electrode (for 105 F g^−1^ at 1 A g^−1^), the as-prepared rGO/PANI/PVA produces a high specific capacitance of 174 F g^−1^ at 1 A g^−1^. At low frequencies, the EIS curve of 0.2% rGO/PANI/PVA has the largest slope, indicating its ideal capacitive behavior with quick ion diffusion and minimal resistance.^120^ Nanosheet-like ZnCo_2_O_4_@N-doped GO/PANI composites were prepared for supercapacitor applications using a CP such as PANI.^121^ The fabricated ZnCo_2_O_4_@N-GO/PANI nanocomposites demonstrated outstanding stability performance with 96.4% capacitance allowance after 10 000 cycles and a specific capacitance of 720 F g^−1^ at a scan rate of 10 mV s^−1^ in 3 M KOH,^121^ making the PANI-incorporated composite a viable catalytic material for use in supercapacitors. For use in supercapacitors, another highly conducting graphite electrode material of MnS/GO/PANI was made using PANI.^122^ When GO was added to pure MnS, the average pore size, surface area, and specific capacitance all increased. Additionally, adding PANI to MnS/GO increases the interfacial contact between the various pore-sized nanoparticles, resulting in improved specific capacitance. According to the GCD tests, the MnS/GO/PANI nanocomposite's specific capacitance was 773 F g^−1^ at a current density of 1 A g^−1^. At a greater current density, the specific capacitance was 484 F g^−1^ at 3.8 A g^−1^. For MnS/GO/PANI nanocomposites, the specific capacitance measured by CV is 822 F g^−1^ at 10 mV s^−1^ and 315 F g^−1^ at 200 mV s^−1^.^120^ Favorable electroactive nanocomposites are a suitable choice for an affordable, future-generation HPS application because of the synergistic effects that can be achieved without damaging the metal sulphide nanostructure. Zhou *et al.* conducted a significant study on the function of various conductive polymers in supercapacitors using a ZnS/rGO/conductive polymer ternary composite electrode.^97^ In the three-electrode and two-electrode setup, ZnS/rGO/PANI demonstrated specific capacitances of 1045.3 F g^−1^ and 722 F g^−1^ at a current density of 1 A g^−1^. The energy density of the two-electrode device is 349.7 W h kg^−1^, and its power density is 18 kW kg^−1^. The fabricated electrode's cycling stability retention is 160% in a 3-electrode system and 76.1% in a 2-electrode arrangement.^97^

### 4.5. Nanocomposites of GO with other materials

GO-based nanocomposites prepared with different materials like metal carbide metal sulphide show great promise owing to their bulky surface area, excellent conductivity, pseudocapacitive properties, and so on. These nanocomposites have better electrochemical performance, including increased cycling stability and specific capacitance, especially when GO is combined with transition metal carbides. Further, the high specific capacitance and pseudocapacitive nature of the produced electrode can be attributed to the presence of metal phosphides.

Two distinct 2D materials, namely reduced graphene oxide (rGO) and titanium carbide MXene (Ti_3_C_2_T_*x*_), have demonstrated encouraging outcomes for supercapacitor applications because of their high conductivity, flake-like shape, and ability to intercalate ions or molecules for charge storage. Following shear mixing, a heterostructure of Ti_3_C_2_T_*x*_ and rGO self-assembled to produce the rGO : Ti_3_C_2_T_*x*_ composites.^123^ The fabricated rGO : Ti_3_C_2_T_*x*_ composite was employed as a supercapacitor electrode. The voltage window was widened to some extent by the incorporation of rGO to Ti_3_C_2_T_*x*_. Furthermore, a rise in the capacitance value was noted as a result of these materials' synergistic action. An electrode film prepared using rGO (1 weight percent) and Ti_3_C_2_T_*x*_ (99 weight percent) exhibited a capacitance up to 254 F g^−1^ at 2 mV s^−1^ and 193 F g^−1^ at 100 mV s^−1^.^123^ In another work, graphene (G) nanoflakes (NFs) were synthesized *via* a two-step process using tungsten carbide (WC) nanoparticles as the electrode for supercapacitors.^124^ Together with the electrical double-layer characteristic, the CV scans showed that a pseudocapacitive contribution governed the charging process. At 1 mV s^−1^, a specific capacitance of 1009.52 F g^−1^ was determined, and following recurrent cycles at 20 A g^−1^ for 2000 cycles, the retention improved by 106%. This approach for the formation of WC@GNFs may be a straightforward way to assess how well a number of similar transition metal carbides perform in relation to supercapacitors.^124^ Li *et al.* described the scalable production of vanadium carbide/graphene electrodes for flexible and high-energy micro supercapacitors (MSCs).^125^ One of the biggest challenges facing flexible MSCs with high energy density is their scalable manufacture. Using laser scribing on ammonium meta-vanadate/GO (NH_4_VO_3_/GO), the authors showed how to create vanadium carbide/rGO (V_8_C_7_/rGO) MSCs. Excellent electrochemical performance is bestowed upon V_8_C_7_/rGO MSCs by their well-defined architecture. These devices have an 11-fold larger areal capacitance than rGO MSCs, with a maximum value of 49.5 mF cm^2^. The power density and volumetric energy density can reach 401 mW cm3 and 3.4 mWh cm^3^, respectively, which is comparable to the majority of reported MSCs and commercially available energy storage devices.^125^ Furthermore, the long cycling life and exceptional flexibility and integrability of V_8_C_7_/rGO MSCs make them promising for real-world applications.

Due to the strong electrochemical reaction activities, transition metal phosphides (TMPs) have been extensively researched as supercapacitor electrodes. In general, TMPs' low conductivity and significant volume fluctuations in electrochemical processes have limited their practical use. Nickel phosphide-containing Ni_2_P-Ni@NC@G composites were created by fabricating and loading a nitrogen-doped carbon (NC)-coated Ni_2_P-Ni hybrid into a favorably conductive graphene network.^126^ The composites had improved rate performance and superior electrode reaction kinetics due to the continuous electron transport channels provided by the highly conductive graphene, NC coating layer, and Ni nanoparticles. Additionally, the well-decorated Ni particles and flexible graphene sheets with Ni_2_P can efficiently shield the damaging stress in electrochemical reactions to maintain a cohesive electrode structure. These advantageous characteristics enabled this composite to exhibit better capacitive and lithium storage performance. This composite reveals an elevated capacitance of 2335.5 F g^−1^ at 1 A g^−1^ and a sharp capacitance retention of 86.4% after 2000 cycles when utilized as an electrode for SCs.^126^ Additionally, asymmetrical supercapacitors (ASCs) were fabricated, exhibiting an impressive energy density of 53.125 Wh kg^−1^ and a power density of 3750 Wh k g^−1^.^126^ Consequently, additional TMP components for systems that store energy could be produced. Utilizing metal phosphide, Chen *et al.* also created a simple and inexpensive technique for creating nickel–cobalt phosphide/multilayer graphene (MLG) (NiCoP/MLG) as an HPS electrode.^98^ Upon testing as a material for supercapacitor electrodes, the resultant porous flower-like NiCoP/MLG showed exceptional electrochemical performance in terms of endurance at elevated current density, continuing action with 91.7% capacitance retention after more than 3000 cycles at a current density of 5 A g^−1^ and a specific capacitance of 1419.6 F g^−1^ at 1 A g^−1^. Consequently, NiCoP/MLG as prepared may be applied as an electrode for HPSs. Excellent electrochemical features were achieved through the design and preparation of novel nickel cobalt phosphide nanowires on 2D-GO nanosheets (GO@NiCoP).^95^ In this case, GO acted as the conductive channel to increase the composite conductivity and supplied a large number of functional groups that included oxygen to coordinate with NiCoP metal cations, thereby increasing the overall structural stability. By utilizing these materials, GO@NiCoP demonstrated an elevated cycling stability of 104.88% and an enhanced specific capacitance of 1125 F g^−1^ (155 mA h g^−1^) at 2 A g^−1^. Interestingly, because of its stable structure and superior conductivity, the GO@NiCoP demonstrated an outstanding rate capability of 84.09% capacitance retention at 20 A g^−1^.^95^ In summary, the exceptional electrochemical capability of the electrode offers a fresh viewpoint on how to increase the electrode materials' rate capacity through the synergistic impact of the outer and inner components.

## 5 Summary and future perspectives

GO-based nanocomposites own a specialized market in supercapacitor research. The GO sheet is not an active material but a conductive, high-area, chemically addressable scaffold on which pseudocapacitive oxides, conducting polymers, and heteroatom dopants are organized. This distinction matters because it reframes the central design question. The problem is not how to increase the intrinsic capacitance of a single component but how to couple a fast electron-conducting network to a high-density redox reservoir without sacrificing one for the other. This review argues that the morphology, dictated by the synthesis route, is the variable that governs this coupling, and that CVD, hydrothermal growth, microwave reduction, and *in situ* oxidation should be understood as tools for engineering the pore architecture and interfacial contact rather than as interchangeable preparation methods.

Furthermore, the primary limitations do not carry equal importance. In many cases, the most pronounced performance gap appears when a material that performs well in a thin laboratory electrode is transferred to a thicker and more practically loaded electrode. Capacitance can drop sharply as mass loading increases because ion transport through the thick electrode becomes slow. At this stage, improving conductivity alone is no longer enough. Restacking GO sheets worsens this condition by closing pores and reducing the surface available to the electrolyte. Spacers, molecular pillars, and crumpled structures can help overcome this problem, but they often introduce another defect, such as lower conductivity or lower packing density. For this reason, mass loading and restacking should be treated as associated problems and addressed before further improving intrinsic capacitance. Degradation of pseudocapacitive components through swelling, dissolution, or structural changes remains a critical concern; however, in many systems, this issue manifests only once transport limitations have been mitigated.

Future efforts should be directed toward these changes. Thick electrodes need to be designed, so that ion transport remains efficient even if the areal mass increases. Vertically aligned pores, freeze-cast structures, and laser-patterned conductive frameworks are attractive because they can shorten the diffusion paths without requiring an extremely thin electrode. Restacking also needs a more permanent solution. Covalently anchored spacers may be useful to maintain the interlayer separation without creating excessive resistance. Once transport and structural accessibility are optimized, the research focus can shift more effectively toward cycle life. Thin conformal redox layers and more stable charge-storing phases, including MXene-based hybrids and metal nitrides, may be highly beneficial. Processing also remains a practical concern. Hydrazine reduction and harsh acid treatments hamper large-scale production, whereas electrochemical and greener reduction methods are more favourable for manufacturing, particularly if the carbon-to-oxygen ratio can be controlled reproducibly.

A substantial commercial gap remains. Most studies still report performance at the material level or in three-electrode configurations, while far fewer studies have examined complete two-electrode cells under realistic conditions. Data on packaged device are still rarely available. Parameters such as full-cell energy density, self-discharge, practical mass loading, and long-term cycling are therefore much less established than the available literature on specific capacitance. Activated carbon remains the commercial reference because it is inexpensive, scalable, and already supported by mature manufacturing. GO-based composites have not yet shown a convincing advantage at that level. The field would therefore benefit more from shifting its evaluation metrics rather than from continuing to compete mainly on record capacitance values. Practical loading, device-level energy and power density, cost per farad, self-discharge, and stability over tens of thousands of cycles are of more importance. The transition of GO-based composites from laboratory research to practical applications will depend largely on their performance under the discussed conditions.

## Author contributions

Mohammad Mizanur Rahman Khan: conceptualization, methodology, investigation, validation, writing – original draft. Md Mahamudul Hasan Rumon: investigation, validation, data curation, writing – review & editing. Jae-Ho Jeong: investigation, validation, data curation, writing – review & editing.

## Conflicts of interest

The authors declare that they have no known competing financial interests or personal relationships that could have appeared to influence the work reported in this paper.

## Data Availability

No primary research results, software, or codes have been included, and no new data were generated or analyzed as part of this review.
